# Natural Killer Cell Receptors and Ligands Are Associated With Markers of HIV-1 Persistence in Chronically Infected ART Suppressed Patients

**DOI:** 10.3389/fcimb.2022.757846

**Published:** 2022-02-10

**Authors:** Geoffrey T. Ivison, Elena Vendrame, Giovanny J. Martínez-Colón, Thanmayi Ranganath, Rosemary Vergara, Nancy Q. Zhao, Maureen P. Martin, Sean C. Bendall, Mary Carrington, Joshua C. Cyktor, Deborah K. McMahon, Joseph Eron, R. Brad Jones, John W. Mellors, Ronald J. Bosch, Rajesh T. Gandhi, Susan Holmes, Catherine A. Blish, The ACTG 5321 Team

**Affiliations:** ^1^ Department of Medicine, Division of Infectious Diseases and Geographic Medicine, Stanford University School of Medicine, Stanford, CA, United States; ^2^ Department of Pathology, Stanford University School of Medicine, Stanford, CA, United States; ^3^ Program in Immunology, Stanford University School of Medicine, Stanford, CA, United States; ^4^ Basic Science Program, Frederick National Laboratory for Cancer Research, National, Cancer Institute, Frederick, MD, United States; ^5^ Laboratory of Integrative Cancer, Immunology, Center for Cancer Research, National Cancer Institute, Bethesda, MD, United States; ^6^ Ragon Institute of Massachusetts General Hospital (MGH), Massachusetts Institute of Technology (MIT), and Harvard, Boston, MA, United States; ^7^ Division of Infectious Diseases, University of Pittsburgh, Pittsburgh, PA, United States; ^8^ Department of Infectious Diseases and Microbiology, University of Pittsburgh Graduate School of Public Health, Pittsburgh, PA, United States; ^9^ Division of Infectious Diseases, University of North Carolina, Chapel Hill, NC, United States; ^10^ Division of Infectious Diseases, Department of Medicine, Weill Cornell Medicine, New York, NY, United States; ^11^ Center for Biostatistics in AIDS Research, Harvard TH Chan School of Public Health, Boston, MA, United States; ^12^ Division of Infectious Diseases, Massachusetts General Hospital, Harvard Medical School, Boston, MA, United States; ^13^ Center for AIDS Research, Harvard University, Boston, MA, United States; ^14^ Department of Statistics, School of Humanities and Sciences, Stanford University, Stanford, CA, United States; ^15^ Chan Zuckerberg Biohub, San Francisco, CA, United States

**Keywords:** human immunodeficiency virus (HIV), HIV latency, natural killer cells, natural killer cell receptor ligands, HIV cure

## Abstract

The latent HIV-1 reservoir represents a major barrier to achieving a long-term antiretroviral therapy (ART)-free remission or cure for HIV-1. Natural Killer (NK) cells are innate immune cells that play a critical role in controlling viral infections and have been shown to be involved in preventing HIV-1 infection and, in those who are infected, delaying time to progression to AIDS. However, their role in limiting HIV-1 persistence on long term ART is still uncharacterized. To identify associations between markers of HIV-1 persistence and the NK cell receptor-ligand repertoire, we used twin mass cytometry panels to characterize the peripheral blood NK receptor-ligand repertoire in individuals with long-term antiretroviral suppression enrolled in the AIDS Clinical Trial Group A5321 study. At the time of testing, participants had been on ART for a median of 7 years, with virological suppression <50 copies/mL since at most 48 weeks on ART. We found that the NK cell receptor and ligand repertoires did not change across three longitudinal samples over one year—a median of 25 weeks and 50 weeks after the initial sampling. To determine the features of the receptor-ligand repertoire that associate with markers of HIV-1 persistence, we performed a LASSO normalized regression. This analysis revealed that the NK cell ligands CD58, HLA-B, and CRACC, as well as the killer cell immunoglobulin-like receptors (KIRs) KIR2DL1, KIR2DL3, and KIR2DS4 were robustly predictive of markers of HIV-1 persistence, as measured by total HIV-1 cell-associated DNA, HIV-1 cell-associated RNA, and single copy HIV-RNA assays. To characterize the roles of cell populations defined by multiple markers, we augmented the LASSO analysis with FlowSOM clustering. This analysis found that a less mature NK cell phenotype (CD16^+^CD56^dim^CD57^-^LILRB1^-^NKG2C^-^) was associated with lower HIV-1 cell associated DNA. Finally, we found that surface expression of HLA-Bw6 measured by CyTOF was associated with lower HIV-1 persistence. Genetic analysis revealed that this was driven by lower HIV-1 persistence in HLA-Bw4/6 heterozygotes. These findings suggest that there may be a role for NK cells in controlling HIV-1 persistence in individuals on long-term ART, which must be corroborated by future studies.

## Introduction

For over two decades, it has been possible for patients to achieve undetectable HIV-1 viral loads, using modern anti-retroviral therapies (ART) (Perelson et al., 1997). Numerous studies since then have demonstrated that patients who are able to strictly adhere to their ART regimens and maintain viral suppression represent negligible risk for sexual transmission of HIV-1 (LeMessurier et al., 2018). While this represents a tremendous victory for public health, it is not yet a complete success. Replication-competent proviruses persist in the genomes of infected CD4^+^ T-cells (Walker et al., 1997; Chun et al., 1998; Chavez et al., 2015; Pardons et al., 2019), as well as potentially other cell types (Eisele and Siliciano, 2012; Kuo and Lichterfeld, 2018). If ART is stopped these latent viruses can cause a rebound of viremia within weeks (Hamlyn et al., 2012; Eisinger et al., 2019). Although rapid post-exposure prophylaxis is able to limit the establishment of this latent reservoir (Sultan et al., 2014; Bamford et al., 2017; Iloanusi et al., 2019), once established it becomes extremely tenacious with an estimated half-life of between 4-13 years with consistent ART treatment (Siliciano et al., 2003; Gandhi et al., 2017). Eliminating the latent HIV-1 reservoir remains the major barrier to achieving an HIV-1 long-term remission or cure.

The factors that control the size of the latent HIV-1 reservoir and HIV-1 persistence on long-term ART remain poorly understood. The most well-established association is that patients that start treatment during the acute phase of infection have lower levels of HIV-1 persistence (Strain et al., 2005; Hocqueloux et al., 2010; Buzon et al., 2014; Bachmann et al., 2019). A previous study on patients enrolled in the AIDS Clinical Trial Group (ACTG) study 5321 found no association between markers of immune activation or inflammation and markers of HIV-1 persistence, but that higher pre-ART plasma HIV-1 RNA levels predicted greater on-ART HIV-1 persistence (Gandhi et al., 2017). Viral sequence analysis has shown that >70% of the viruses found in the reservoir in patients on long-term ART are the same as the viruses found early in infection, suggesting that the reservoir is seeded at the initiation of ART, and that events later in infection likely play a limited role in shaping the reservoir (Abrahams et al., 2019). T-cell responses targeting the HIV protein Nef have been shown to correlate with HIV-1 persistence (Thomas et al., 2017), as have variants of Nef (Omondi et al., 2019). Anti-HIV-1 antibody levels have a positive correlation with HIV-1 persistence (Keating et al., 2019). CD45 expression on CD4^+^ and CD8^+^ T-cells has also been shown to correlate with HIV-1 persistence (Petkov et al., 2020). Much of the possible relationship between the persistence of HIV-1 infected cells and the immune system has not yet been explored.

The role of Natural Killer (NK) cells in controlling HIV-1 has been increasingly appreciated (Wilk and Blish, 2018). While it was initially thought that HIV-1 eluded NK cell responses by selectively downregulating only certain HLA class I molecules (Cohen et al., 1999), leaving the primary KIR ligand, HLA-C, intact, this was later found to be a result of using lab adapted strains of HIV-1 (Apps et al., 2016). In fact, NK cells do kill HIV-1 infected cells *in vitro* (Boudreau et al., 2016). There is a well-established genetic association, where individuals infected with HIV-1 possessing certain combinations of the NK cell inhibitory receptor KIR3DL1 and its ligand HLA-Bw4 are slower to progress to AIDS (Martin et al., 2007). Having a less diverse and more flexible NK cell repertoire has also been associated with reduced HIV-1 acquisition in a cohort of pregnant and post-partum women in Africa (Strauss-Albee et al., 2015), and the NK cell phenotype is broadly altered in highly HIV-exposed seronegative individuals in another African cohort (Zhao et al., 2020). Reduced HIV acquisition was also associated with more active NK cells in a cohort of HIV exposed-uninfected people who inject drugs (Scott-Algara et al., 2003). The NK cell repertoire is also profoundly altered by HIV-1 infection, including expansion of a CD56^-^CD16^+^ subpopulation (Hu et al., 1995; Alter et al., 2005; Mavilio et al., 2005), downregulation of the activating receptors NKp30 and NKp46 (Mavilio et al., 2003), and upregulation of the inhibitory receptor TIGIT (Yin et al., 2018; Vendrame et al., 2020). Another crucial element of the NK cell response is the expression of NK receptor ligands on other cell populations. However, very little research has been done to investigate the role NK receptor ligands in HIV-1 infection, other than HLA class I. No approach has yet characterized the association of NK cell receptors and ligands with markers of HIV-1 persistence.

In this study, we sought to understand the role of NK receptors and ligands in relation to HIV-1 persistence. We evaluated a cohort of 50 participants enrolled in the ACTG 5321 study, with samples from three separate time points, all on long term ART. We used twin mass cytometry (CyTOF) panels measuring both NK receptors and ligands, and combined these data with other measurements of immune activity to gain a more complete picture of the NK cell receptor-ligand repertoire in relationship to markers of HIV-1 persistence in individuals on long-term ART.

## Materials and Methods

### Study Population

﻿We evaluated a longitudinal cohort of participants with chronic HIV-1 infection who initiated ART in AIDS Clinical Trials Group (ACTG) trials for treatment-naive people and had sub- sequent follow-up while continuing to receive ART (ACTG A5321) (Gandhi et al., 2017). Participants had plasma HIV-1 RNA levels <50 copies/mL by commercial assays starting at week 48 of ART and at all subsequent time points with no reported ART interruptions. Pre-ART measurements of plasma viral load and CD4 cell counts were available. We performed NK cell receptor and ligand measurements on cryopreserved peripheral blood mononuclear cells (PBMCs) from three on-ART timepoints (median of 7 years on ART, 25 weeks later, and 50 weeks later).

### Virologic Assays

Cell-associated HIV-1 DNA (CA-DNA) and cell-associated HIV-1 RNA (CA-RNA) were measured by quantitative PCR (qPCR) in PBMC samples using methods that have been previously published (Hong et al., 2016). Plasma HIV-1 RNA by single-copy assay (SCA) was measured using methods that have been previously published (Cillo et al., 2014). ﻿Primers and probes used for qPCR of CA-DNA, CA-RNA and plasma HIV-1 RNA were identical (Cillo et al., 2014; Hong et al., 2016). ﻿CA-DNA and CA-RNA values per million CD4^+^ T- cells were calculated by dividing the total HIV-1 DNA or RNA copies/million PBMCs [normalized for CCR5 copies measured by qPCR as published (Hong et al., 2016)] by the CD4^+^ T-cell percentage (x 0.01) reported from the same specimen date or from a CD4^+^ T-cell percentage imputed using linear interpolation from specimen dates before and after the CA-DNA or CA-RNA results.

### Assays for Soluble Markers of Inflammation

Plasma concentrations of interleukin-6 (IL-6), C-X-C motif chemokine ligand 10 (CXCL10), neopterin, tumor necrosis factor-α (TNF-α), high sensitivity C reactive protein (hsCRP), soluble CD14 (sCD14), and soluble CD163 (sCD163) were quantified using enzyme-linked immunosorbent assay (ELISA) kits per manufacturer’s instructions (R&D, Minneapolis, MN).

### IFN-γ ELISPOT

T-cell reactivity to peptides was determined by ELISPOT as described previously (Thomas et al., 2017). Briefly, ﻿consensus HIV-1 Clade B 15 amino acid peptides overlapping by 11 amino acids spanning the entire HIV genome were obtained from the NIH AIDS Research and Reference Reagent Program. ﻿A CMV-pp65 peptide pool and an EBV BZLF-1 peptide pool were obtained from JPT peptide technologies. 96 well plates were coated overnight with anti-IFNγ antibody and then washed. PBMCs were thawed and then added to wells with peptide pools, then incubated for 18 hours. Plates were then washed again and sequentially stained with biotinylated secondary antibody, streptavidin-alkaline phosphatase, and color development solution. Plates were then washed with tween and water and dried overnight. Spots were then counted and spot forming units (sfu)/million PBMC were averaged across two duplicate wells, with negative controls run in triplicate. ﻿A positive response was considered as one which met both of the following two criteria: 1) >50 sfu/million PBMC after background subtraction 2) >2x above background.

### Antibody Conjugation for CyTOF

﻿Antibodies were conjugated using MaxPar x8-labeling kits (Fluidigm, South San Francisco, California, USA). To ensure antibody stability over time, the NK cell panel (**Supplemental Table 1**) was lyophilized into single-use pellets prior to use (Biolyph, Chaska, Minnesota, USA). The ligand panel (**Supplemental Table 2**) was aliquoted into single-use tubes and stored at -80°C.

### Cell Isolation and Staining

Cell isolation and staining for CyTOF was performed as described previously (McKechnie et al., 2020; Vendrame et al., 2020). Briefly, PBMCs were thawed in complete media and counted, then 1 million PBMCs for each participant were kept on ice for ligand panel staining. The remaining PBMCs were used for NK cell purification by negative selection using a human NK Cell Isolation Kit (Miltenyi). PBMCs and isolated NK cells were stained using the viability marker cisplatin (Enzo Life Sciences), as previously described (Fienberg et al., 2012). They were then barcoded with a two-of-four CD45-palladium barcoding scheme generated in-house, using ^102^Pd, ^104^Pd, ^106^Pd, and ^108^Pd. Barcoded samples were pooled and stained with surface antibodies before fixation with 2% paraformaldehyde and permeabilization (eBioscience Permeabilization Buffer). Samples were then stained with intracellular antibodies and incubated in iridium-191/193 intercalator (Fluidigm) for up to a week. Before the analysis by CyTOF, samples were washed and diluted in EQ Four Element Calibration Beads (Fluidigm). Samples were then acquired on a Helios mass cytometer (Fluidigm). These data are available on ImmPort (https://www.immport.org) under study accession SDY1844.

### Statistical Analyses

The open-source statistical language R was used for all statistical analyses (R Core Team, 2020). Data analysis was aided by use of the packages magrittr (Bache and Wickham, 2020), and tidyverse (Wickham et al., 2019). Data were visualized with use of the packages ggplot2 (Wickham, 2016), ggrepel (Slowikowski, 2021), and ggpubr (Kassambara, 2020).

### CyTOF Data Pre-Processing

CyTOF data were bead-normalized (Finck et al., 2013) and then de-barcoded prior to subsequent analyses using the package Premessa (https://github.com/ParkerICI/premessa). NK cell data were first visualized with FlowJo v10.5.3 (Tree Star, Woodburn, OR, USA), while ligand data were visualized with cellEngine (https://cellengine.com/). Gating schemes used to identify NK cells, Monocytes, CD4^+^ T-cells, and CD8^+^ T-cells are shown in **Supplemental Figure 1**. Gated populations were exported for further analysis, and imported into R using flowCore (Ellis et al., 2020). Signal intensities were rounded up to the nearest integer and transformed using the inverse hyperbolic sine (asinh) function with a cofactor equal to 5 to account for heteroskedasticity.

### Dimensionality Reduction

Dimension reduced visualizations of CyTOF data were generated by UMAP and multidimensional scaling (MDS). UMAP embeddings with density correction were calculated on gated populations using the densvis package (Narayan et al., 2021) with default settings and 200 learning epochs. To create MDS embeddings, mean expression of phenotyping markers were calculated for each sample, for each of the four populations. Eight samples were then excluded because any of the four populations had fewer than 200 cells. Euclidean distance was calculated between all points, and classic multidimensional scaling was performed using the cmdscale function.

### LASSO Regression Analysis

LASSO analysis was performed using the package glmnet (Friedman et al., 2010). Mean expression of phenotyping markers from CyTOF data were calculated for each sample from timepoint 1, for each of the four populations. Two samples were then removed because any of the four populations had fewer than 200 cells. CyTOF data were then merged with data from assays for soluble markers of inflammation, ELISPOT data, pre-ART CD4 count, pre-ART CD8 count, pre-ART CD4/CD8 ratio, pre-ART plasma HIV-1 viral load, sex, age, ART regimen at initiation of ART (iART), and years on ART at timepoint 1. These variables were then centered to a mean of zero and scaled to a standard deviation of one, then used as predictors for regression models predicting CA-DNA, CA-RNA, and SCA. Measurements of HIV-1 persistence and ELISPOT data were transformed using the function Log_10_ (x+1). Samples were excluded if data were missing for the respective outcome variable: one sample for CA-DNA, two samples for CA-RNA, and three samples for SCA. The optimal value for the tuning parameter lambda was found using leave-one-out cross validation to determine the value of lambda that provided minimal estimated cross-validation error. The LASSO was considered to have selected a variable if it was present in the model with the minimum cross validated mean squared error. The broom package (Robinson et al., 2021) was used to aid in generating visualizations.

### Clustering Analysis

Unsupervised clustering on gated populations from all three timepoints was performed using the R package CATALYST (Crowell et al., 2020). Samples were excluded if the number of cells in the population being clustered was fewer than 200: seven samples from the NK cells, one sample from the monocytes, and none for the CD4^+^ T-cells and CD8^+^ T-cells. The clustering method in the CATALYST package uses the FlowSOM algorithm (Van Gassen et al., 2015) to generate 100 high-resolution clusters, followed by a metaclustering step with the ConsensusClusterPlus algorithm (Wilkerson and Hayes, 2010). Default parameters were used for clustering, and the number of metaclusters was determined by observing an elbow point in the delta area curve provided by CATALYST, indicating diminishing returns in larger numbers of clusters. The frequency of each cluster for each sample from timepoint 1 were then calculated, centered to a mean of zero, and scaled to a standard deviation of one. These frequencies were then used as predictors for LASSO regression models predicting CA-DNA, CA-RNA, and SCA. Samples were excluded if data were missing for the respective outcome variable: one sample for CA-DNA, two samples for CA-RNA, and three samples for SCA. The optimal value for the tuning parameter lambda was found using leave-one-out cross validation to determine the value of lambda that provided minimal estimated cross-validation error. The LASSO was considered to have selected a variable 1) if it was present in the model with the minimum cross validated mean squared error or 2) if the model with the minimum cross validated mean squared error contained only the intercept, then if it was present in the least stringent reasonable model (within a standard error of the intercept only model) and had a coefficient with absolute value greater than 0.1.

### Calculation of NK Cell Diversity

Diversity was computed based on Boolean expression of 28 markers: NKp30, NKG2C, KIR2DL1, CD94, KIR2DL5, CD62L, KIR2DS4, NKp46, NKG2D, TIGIT, 2B4, FAS_L, Siglec7, CD96, KIR2DL3, CD69, CD2, FcRg, Syk, CD38, DNAM_1, PD1, CD57, NKG2A, NTB_A, KIR3DL1, Perforin, and LILRB1. It was calculated using the Inverse Simpson Index as previously described (Horowitz et al., 2013).

### HLA Genotyping

HLA typing was performed using a targeted next generation sequencing (NGS) method. Briefly, locus-specific primers were used to amplify a total of 25 polymorphic exons of HLA-A & B (exons 1 to 4), C (exons 1 to 5), E (exon 3), DPA1 (exon 2), DPB1 (exons 2 to 4), DQA1 (exon 1 to 3), DQB1 (exons 2 & 3), DRB1 (exons 2 & 3), and DRB3, 4, 5 (exon 2) genes with Fluidigm Access Array (Fluidigm Singapore PTE Ltd, Singapore). The 25 Fluidigm PCR amplicons were pooled and subjected to sequencing on an Illumina MiSeq sequencer (Illumina, San Diego, CA 92122 USA). HLA alleles and genotypes were called using the Omixon HLA Explore (version 2.0.0) software (Omixon, Budapest, Hungary).

### KIR Genotyping

KIR genotyping for the presence or absence of each KIR gene was conducted by PCR with sequence-specific priming (PCR-SSP) as described previously (Martin and Carrington, 2008), with some modifications. Each PCR was conducted in a volume of 5 µL using 5 ng genomic DNA and SYBR Green PCR Master Mix with Platinum Taq (Invitrogen). Presence and absence of specific PCR products is detected by melting curve analysis on the 7900 Real-Time PCR System (Applied Biosystems). An algorithm developed in-house assigns KIR genotypes based on melting curve data. Additional 2DS4 subtyping to detect the null variant, which has a 22bp deletion in exon 5, is performed by size discrimination of the 2DS4 PCR products using the LabChip GX instrument (Caliper).

## Results

### Study Population

﻿The study population consisted of 50 participants with HIV-1 infection who had plasma HIV-1 RNA levels consistently <50 copies/mL as measured by commercial assays at all time points at and after week 48 of ART treatment. The characteristics of the population (summarized in **Table 1**) were notable for the following: median age at first timepoint, 42.5 years; 20% female, median pre-ART plasma HIV-1 RNA of 4.7 log10 copies/mL and median pre-therapy CD4 T-cell count of 256/mm^3^. Regimens at time of ART initiation were non-nucleoside reverse transcriptase inhibitor (NNRTI)-based in 42%, protease inhibitor (PI)- based in 34% and integrase strand transfer inhibitor (InSTI)-based in 18% (all participants on an INSTI received raltegravir). At the first time point that was tested, participants had been on ART for a median of 7 years (interquartile range (IQR), 5–8), and the median CD4+ T-cell count was 651/mm^3^. The second timepoint was taken a median of 25 weeks after timepoint 1, and the third timepoint a median of 50 weeks after timepoint 1.

**Table 1:** Cohort demographics.

Age at first timepoint, median (Q1-Q3), years	42.5 (37.25-51.25)
Sex-Female	20%
Race/Ethnicity:	
White Non-Hispanic	32 (64%)
Black Non-Hispanic	8 (16%)
Hispanic (Regardless of race)	9 (18%)
American Indian, Alaskan Native	1 (2%)
Pre-ART plasma HIV-1 DNA, median (Q1-Q3), log10 copies/mL	4.7 (4.5-5.0)
Pre-ART CD4+ T cell count, median (Q1-Q3)	256 (137-358)
Anti-retrovial regimen at start of therapy:	
NNRTI-based	42%
PI-based	34%
INSTI-based	18%
Other	6%
Years on therapy at first timepoint, median (Q1-Q3)	7.0 (4.5-7.7)
CD4+ T cell count at first timepoint, median (Q1-A3)	651 (519-797)

### Deep Characterization of NK Receptor and Ligand Expression in Patients on Long-Term ART Reveals no Longitudinal Changes

**Figure 1:**
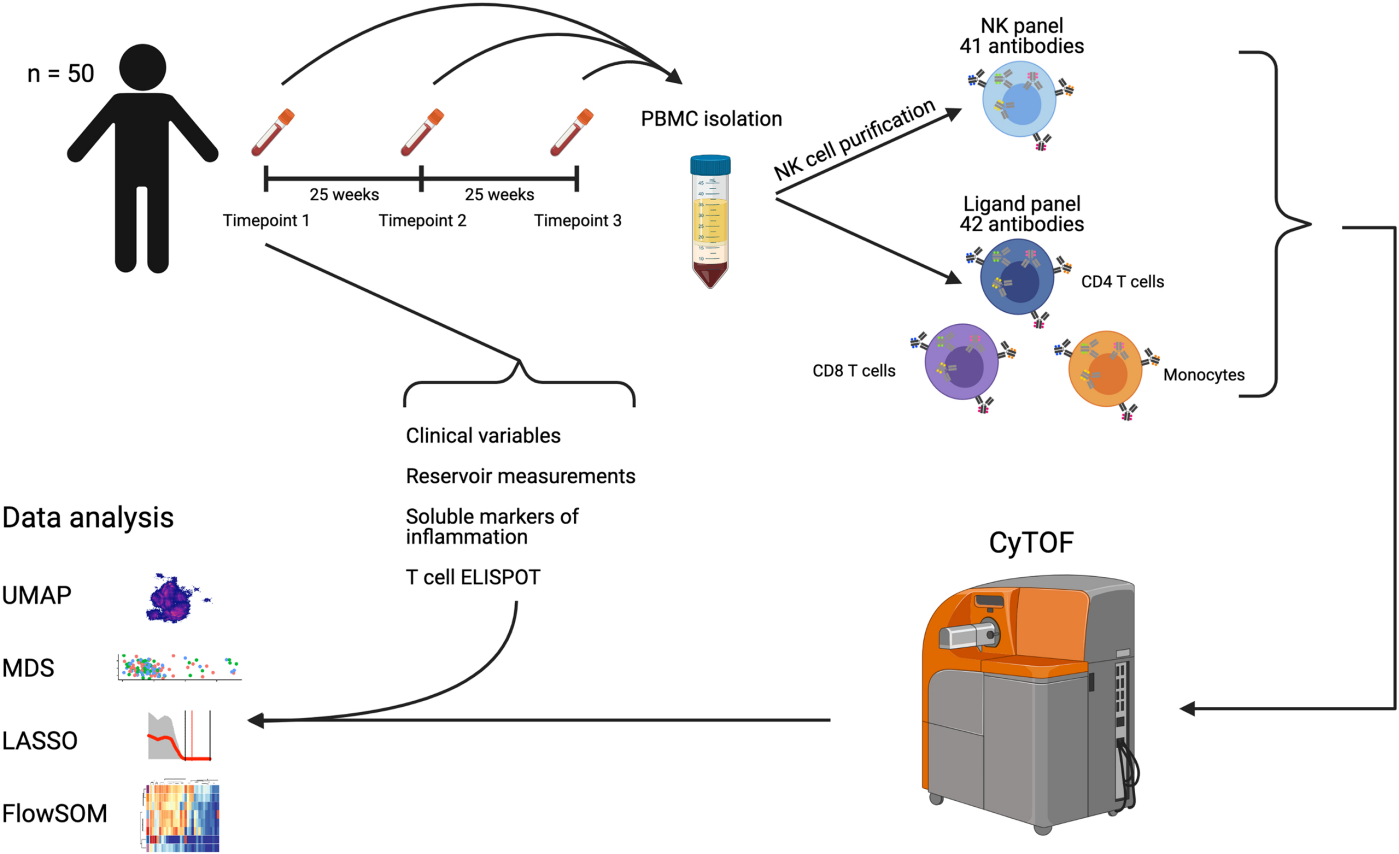
Description of cohort and workflow. We obtained samples from 50 individuals chronically infected with HIV-1 and on long term ART with successful viral suppression, at three timepoints. PBMCs were purified, and cryogenically stored. Samples were then thawed and divided, with a portion of each sample stained with our NK ligand panel of antibodies, and the rest used to purify NK cells, which were then stained with our NK receptor panel of antibodies. Following staining, samples were acquired by CyTOF. Our CyTOF data were analyzed by UMAP, MDS, and FlowSOM. We also obtained virological and immunological measurements from each participant at the first timepoint. These were combined with our CyTOF measurements for analysis by LASSO. Created with Biorender.com.

**Figure 2:**
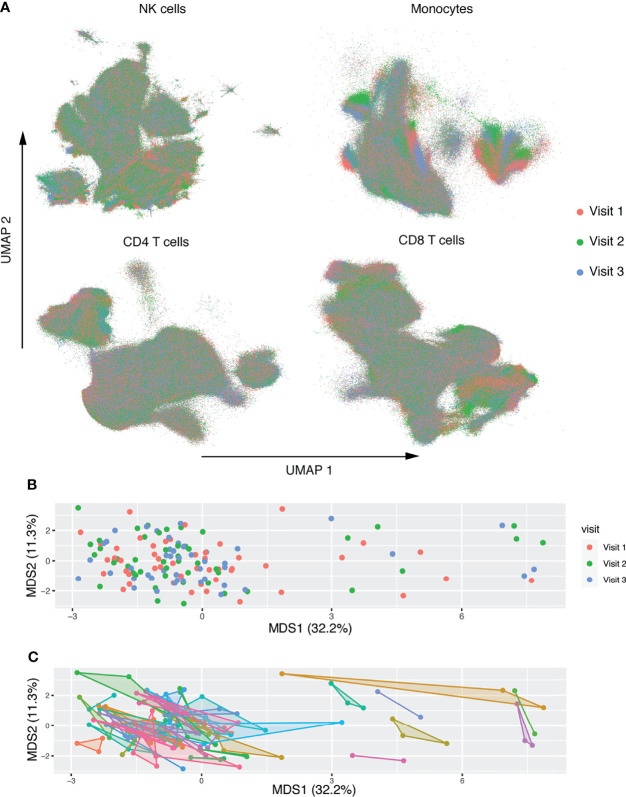
Deep characterization of NK receptor and ligand expression in patients on long term ART reveals no longitudinal changes. **(A)** UMAP embeddings of gated NK cells based on expression of NK receptors, and gated monocytes, CD4^+^ T-cells, and CD8^+^ T-cells based on expression of NK ligands. Colored by week of sample. **(B, C)** MDS embedding, where each point represents the mean expression of NK markers on all NK cells and ligand markers on each population. After filtering for low cell count files, n = 50 participants with n = 142 total samples. In **(B)** points are colored by timepoint. In **(C)** shaded convex hulls connect the points of each participant. Each axis is scaled by the percent variance explained by that axis.

A schematic illustration of our workflow is shown in **Figure 1**. To investigate the longitudinal changes in the NK receptor and ligand repertoire of patients on long-term ART, we used the CyTOF panels in **Supplemental Tables 1, 2** on 50 patient samples from three separate timepoints: a median of 7 years after ART initiation, median of 25 weeks after timepoint 1, and a median of 50 weeks after timepoint 1. Gated populations were then embedded using UMAP and colored by timepoint (**Figure 2A**). This analysis showed no separation of the cell phenotypes by timepoint. Mean values of NK receptor and ligand profiles from each timepoint were also embedded using multidimensional scaling (MDS). This analysis also showed no separation by timepoint (**Figure 2B**) and showed that intra-participant variability was small (**Figure 2C**). A permutation analysis was also performed to quantify intra-participant vs. inter-participant variability, wherein participant labels for each of the samples were randomized and the maximum distance between a pair of samples for each participant was calculated to determine a null distribution. This analysis revealed that intra-participant variability was significantly lower than expected by chance (p < 0.001). Having thus seen that the NK receptor and ligand phenotypes at the three timepoints do not vary significantly from each other, we decided to focus our further analysis only on the first timepoint, which was also the timepoint for which we had measurements of HIV-1 persistence.

### LASSO Normalized Regression Reveals Novel Associations Between Clinical Variables, NK Receptors and Ligands, and Markers of HIV-1 Persistence

**Figure 3:**
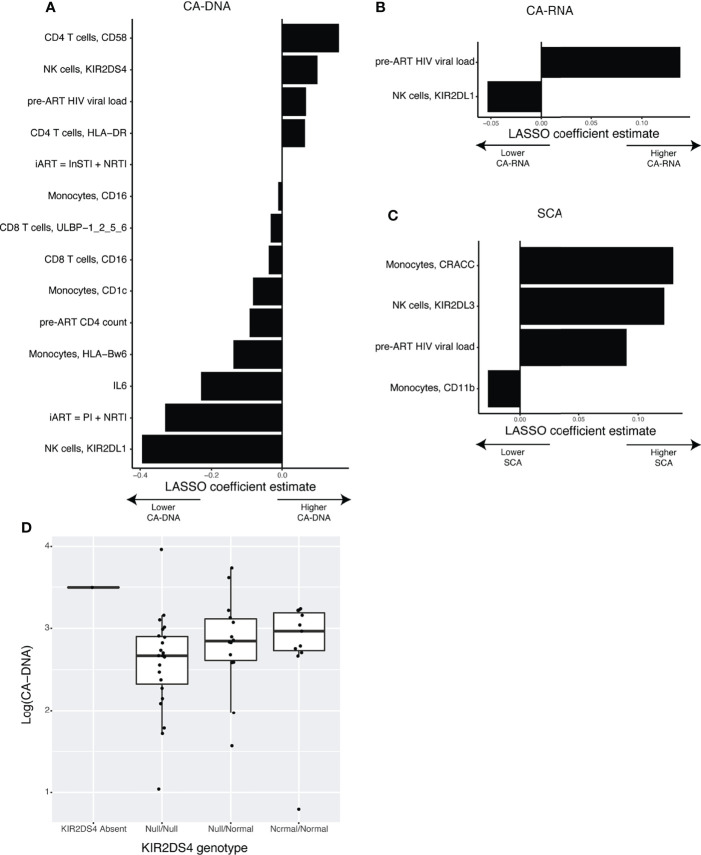
LASSO normalized regression reveals novel associations between clinical variables, NK receptors and ligands, and the HIV-1 latent reservoir. **(A–C)** Plots showing the LASSO coefficient estimates for the optimal models predicting **(A)** CA-DNA (n = 47), **(B)** CA-RNA (n = 46), or **(C)** SCA (n = 45). **(D)** Boxplot showing the association between KIR2DS4 genotype and CA-DNA.

Having a great wealth of data in the form of CyTOF measurements of the NK cell receptor and ligand repertoire, inflammation, T-cell activation, and clinical parameters, we sought to disentangle which factors correlate with markers of HIV-1 persistence. Because the number of parameters available (p = 150) was much higher than our number of study participants (n = 50), we decided to use LASSO normalized regression. This approach is an augmentation of the traditional multivariate regression framework, in which the model is penalized for including more variables, according to a tuning parameter lambda. A larger value for lambda creates a stronger penalty, and therefore selects fewer variables. The optimal value of lambda is the one that includes enough variables to generate a strong prediction, while removing enough variables to reduce overfitting of the model. We determined this optimal value through leave-one-out cross validation, for models predicting CA-DNA (**Figure 3A**), CA-RNA (**Figure 3B**), and SCA (**Figure 3C**). Details of cross validation and variable selection are shown in **Supplemental Figure 2**. This approach was unable to distinguish between contributions of expression level vs percentage positivity for protein expression. Reassuringly, a strong positive predictor for all three markers of HIV-1 persistence was the pre-ART HIV-1 viral load, as has been previously shown for CA-DNA (Allavena et al., 2015) and SCA (Palmer et al., 2008; Riddler et al., 2016). Strong positive predictors for CA-DNA include the CD4 T-cell expression of CD58, while strong negative predictors include NK cell expression of KIR2DL1, initial ART (iART) regimen of a protease inhibitor (PI) and a nucleoside reverse transcriptase inhibitor (NRTI), serum levels of IL-6, pre-ART CD4 count, and Monocyte expression of HLA-Bw6. Negative predictors of CA-RNA include NK cell expression of KIR2DL1. Positive predictors of SCA include Monocyte expression of CRACC and NK cell expression of KIR2DL3, while the only negative predictor was Monocyte expression of CD11b. Univariate associations between every selected variable and its associated outcome are shown in **Supplemental Figure 3** and estimated LASSO coefficients as well as Spearman correlation coefficients are shown in **Supplemental Table 3**. The finding of associations between markers of HIV-1 persistence and expression of KIRs was further evaluated by determining the genotypes of the participants with regards to KIR2DL1, KIR2DL3, and KIR2DS4. We found that all the participants in our cohort possessed at least one copy of KIR2DL1, and the vast majority of the participants possessed at least one copy of KIR2DL3 (n=47) and so we were unable to assess the relationship between KIR genotype and reservoir. We found that participants with or heterozygous for the null allele of KIR2DS4, which is not expressed on the cell surface (Middleton et al., 2007), tended to have lower CA-DNA than donors with the normal variant (**Figure 3D**). This is consistent with the finding from the LASSO that higher KIR2DS4 expression is predictive of higher CA-DNA. Interestingly, the one donor that was KIR2DS4 negative had a relatively high level of CA-DNA, although their CA-DNA was within the range observed for donors homozygous for the null allele of KIR2DS4.

### FlowSOM and LASSO Analysis Reveals an Association Between Less Mature NK Cells and Reduced CA-DNA

**Figure 4:**
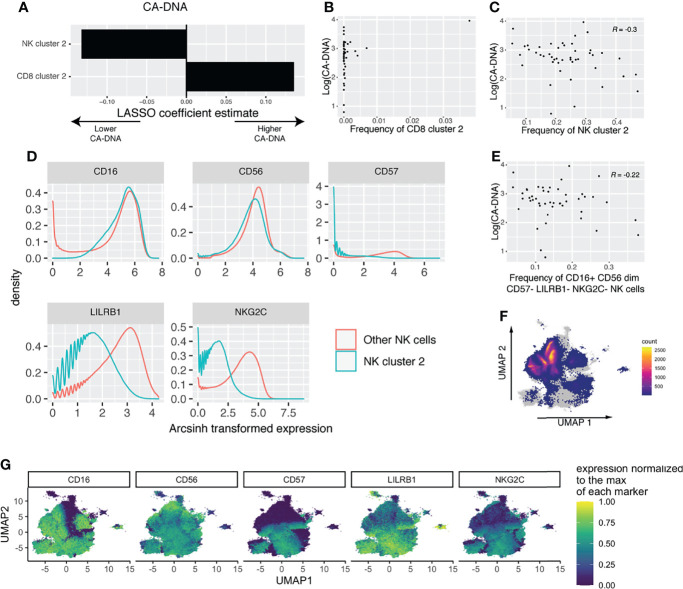
FlowSOM and LASSO analysis reveals an association between immature NK cells and reduced CA-DNA. **(A)** Estimated LASSO coefficients for clusters with large coefficients included in the least stringent model within one standard error of the zero coefficient model (n = 47). **(B)** Scatterplot showing the association between CD8 cluster 2 and CA-DNA. **(C)** Scatterplot showing the association between NK cluster 2 and CA-DNA. **(D)** Histograms showing the phenotype of NK cluster 2 compared to all other NK cells. **(E)** Scatterplot showing the association between the frequency of gated CD16^+^ CD56^dim^ CD57^-^ LILRB1^-^ NKG2C^-^ NK cells and CA-DNA. **(F)** UMAP showing the location of cluster 2 on the total NK cell UMAP, with cells not in cluster 2 shown in grey and the density of cluster 2 shown in color. **(G)** Expression of the markers used to define cluster 2 shown on the UMAP of total NK cells.

Our previous LASSO models might have missed important-cell populations that were defined by multiple markers. To address this, we performed high resolution FlowSOM clustering, followed by ConensusClusterPlus metaclustering (**Supplemental Figure 4**). We then used the frequency of each cluster in each sample as predictors for LASSO models for CA-DNA, CA-RNA, and SCA. The LASSO model for CA-DNA identified one NK cell cluster and one CD8 T-cell cluster as predictive (**Figure 4A**). However, scrutiny of the associations between cluster frequency and CA-DNA revealed that the association found with the CD8 T-cell cluster was driven by outliers (**Figure 4B**), while the association between NK cluster 2 and CA-DNA was more robust (**Figure 4C**). NK cell cluster 2 has an immature conventional NK cell phenotype, CD16^+^ CD56^dim^ CD57^-^ LILRB1^-^ NKG2C^-^ (**Figures 4D, F, G**). Manual gating analysis for NK cells matching this phenotype (**Supplemental Figure 5**) was able to recover a population of NK cells that also has a negative correlation with CA-DNA (**Figure 4E**). It has been shown that CD57^-^ NK cells correlate with lower diversity of the NK cell repertoire, which in turn is associated with lower rates of HIV acquisition (Strauss-Albee et al., 2015). We subsequently examined the relationship between HIV diversity and markers of HIV persistence, but we found no association (**Supplemental Figure 6**). Given the association between NK cell maturity and expression of KIRs and NKG2A (Björkström et al., 2010), we examined expression of these markers on NK cluster 2. Interestingly, we observed slightly lower expression of both NKG2A (suggesting maturity) and KIRs (suggesting immaturity), and no change in expression of perforin (**Supplemental Figure 7A**). This makes it harder to neatly classify this population as either mature or immature. Considering that several of the markers we used to define this population are associated with CMV serostatus, we were interested to find whether the canonical NK memory population (NKG2C^hi^ CD57^+^) had any association with CA-DNA. However, we found no such association (**Supplemental Figure 7B**). While we did not have CMV serostatus on these specific individuals, previous results from another subset of ACTG5321 with similar demographics to our cohort found that participants were 94% CMV seropositive (Hodowanec et al., 2019), so we are likely underpowered to examine the relationship between CMV serostatus and NK cell phenotype or HIV persistence. LASSO analysis for CA-RNA revealed no predictive clusters (**Supplemental Figures 8C, D**). LASSO analysis for SCA found several predictive clusters, but each of the associations were driven by outliers (**Supplemental Figures 8E–G**). We also examined the relationship between NK cluster frequencies and pre-ART HIV viral load and found no strong associations (**Supplemental Figure 9**).

### Association Between HLA-Bw4/6 Genotype and Markers of HIV-1 Persistence

**Figure 5:**
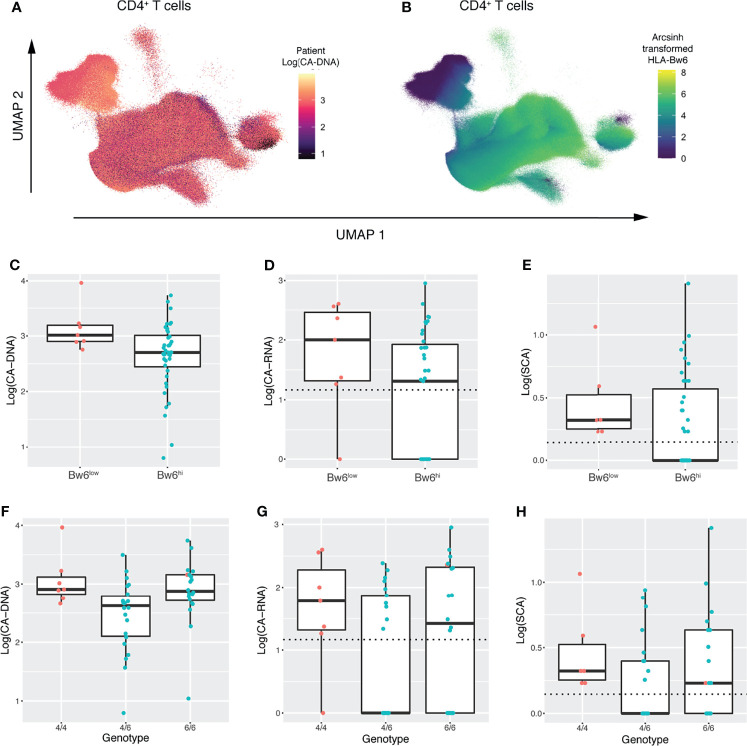
UMAP embeddings and genetic analysis reveal an association between HLA-Bw4/6 phenotype/genotype and the latent HIV-1 reservoir. **(A)** UMAP embedding of CD4+ T-cells colored by participant CA-DNA at entry. **(B)** UMAP embedding of CD4+ T-cells colored by HLA-Bw6 expression **(C–E)** Boxplots showing **(C)** CA-DNA, **(D)** CA-RNA, and **(E)** SCA in participants with low vs. high HLA-Bw6 expression as measured by CyTOF. Dashed horizontal lines indicate the limit of detection for each assay. Points are colored by Bw6 hi vs. low as determined in **Supplemental Figure 11** **(F–H)** Boxplots showing **(F)** CA-DNA, **(G)** CA-RNA, and **(H)** SCA in participants with Bw4/4, Bw4/6, and Bw6/6 genotypes. Dashed horizontal lines indicate the limit of detection for each assay. Points are colored by Bw6 hi vs. low as measured by CyTOF.

Having found an association between expression of HLA-Bw6 and CA-DNA by LASSO analysis, we were interested in investigating this finding further. Coloring the CD4 T-cell UMAP embedding by each marker and by markers of HIV-1 persistence revealed a correspondence between samples with higher CA-DNA and lower HLA-Bw6 expression (**Figures 5A, B** and **Supplemental Figure 11**). Similar visualizations revealed the same association in the UMAP for CD8 T-cells (**Supplemental Figure 12**) and monocytes (**Supplemental Figure 13**), while no associations were readily apparent in the UMAP for NK cells, which does not include HLA-Bw6 (**Supplemental Figure 10**). Separating participants into Bw6 high and low by mean expression of HLA-Bw6 (**Supplemental Figure 14**) revealed an association between Bw6 expression on CD4 T-cells and CA-DNA, CA-RNA, and SCA (**Figures 5C–E**). Similar associations were observed in CD8 T-cells and Monocytes (not shown). To determine whether this phenotype was corroborated at the genetic level, HLA typing was performed on these participants. It was revealed that participants that were HLA-Bw4/6 heterozygous had the lowest CA-DNA, CA-RNA, and SCA measurements (**Figures 5F–H**). Since the Bw6 hi population observed on CyTOF consists of both Bw4/6 heterozygotes and Bw6/6 homozygotes, this is consistent with the results observed on CyTOF. As heterozygotes for HLA-Bw4/Bw6 are composed of allotypes that are generally more distinct from one another relative to those allotypes present in the HLA-Bw4/Bw4 or Bw6/Bw6 groupings, these data may suggest that greater breadth in peptide repertoire among HLA-Bw4/Bw6 heterozygotes is advantageous through improved T-cell-mediated response to HIV-1. Consequently, we looked at the association between T-cell responses to peptides *via* IFNγ ELISPOT. However, we observed no improved response to peptides from peripheral blood T-cells in Bw4/6 heterozygotes (**Supplemental Figure 15A**). As suggested by the LASSO analyses, there was also no association between ELISPOT responsiveness and markers of HIV-1 persistence (**Supplemental Figures 15B–D**).

## Discussion

Understanding the mechanisms controlling the latent HIV-1 reservoirs in patients on long-term ART will inform HIV-1 cure strategies. NK cells have been implicated in control of HIV-1 in long-term non-progressors and highly exposed seronegative individuals. In this study, we deeply profiled the NK receptor and ligand repertoire, in combination with measurements of inflammation, T-cell activity and markers of HIV-1 persistence, to gain a more complete picture of the NK cell receptor-ligand repertoire in relation to HIV-1 persistence in individuals on long-term ART. We found associations between the NK cell receptor-ligand repertoire and markers of HIV-1 persistence, which suggest that NK cells may play a role in determining the level of HIV-1 reservoir.

While much work has characterized the role of NK cells in early infection, no longitudinal studies have characterized how the NK cell receptor-ligand repertoire changes over time in chronically infected individuals. We observed minimal change in the NK cell receptor and ligand repertoire in our cohort over the course of approximately one year on ART. This is consistent with a longitudinal study of healthy individuals, which found minimal changes in the NK cell repertoire over the course of six months (Strauss-Albee et al., 2015). This suggests that in long term ART without spikes of viremia, the NK cell response reaches a steady state, as has also been shown for T-cell responses (Xu et al., 2019).

We found several immune, virological, and clinical predictors of the HIV-1 persistence through our LASSO analysis. Most consistently, the LASSO found that pre-ART HIV-1 plasma viremia is a predictor of all three markers of HIV-1 persistence. This is corroborated by an earlier study that found an association between pre-ART viremia and CA-DNA (Allavena et al., 2015). Conversely, a lower pre-ART CD4 count correlated with higher CA-DNA. Both the association between higher pre-ART viremia and lower pre-ART CD4 count with CA-DNA were also corroborated by a study (Bachmann et al., 2019) which followed a cohort of 1057 HIV-infected patients on ART for a median of 5.4 years. Taken together, these findings suggest that chronic infection and higher viremia prior to initiation of ART sets conditions that enable long-term persistence in the viral reservoir.

We also found in our cohort an effect of initial ART (iART) regimen, where regimens containing an integrase strand transfer inhibitor (InSTI) in combination with a nucleoside reverse transcriptase inhibitor (NRTI) were associated with higher CA-DNA, while a protease inhibitor (PI) plus an NRTI was associated with lower CA-DNA. However, a recently published study using a larger portion of the A5321 cohort (Cyktor et al., 2021) found no association between iART and CA-DNA. We therefore regard this finding to be a result of a random artifact of sampling.

We also found a negative association between IL-6 and CA-DNA, which an earlier, larger study on samples from A5321 found was non-significant (Gandhi et al., 2017). Scrutiny of the univariate association between IL-6 and CA-DNA (**Supplemental Figure 6**) suggests that this finding may have been driven by outliers in our data. We suspect that there may be a weak relationship between IL-6 and the HIV-1 persistence on long term ART.

Several NK cell ligands were found to be predictive of HIV-1 persistence, including CD4 T-cell expression of CD58 (predictive of higher CA-DNA) and Monocyte expression of CRACC (predictive of higher SCA). Little is known about how these molecules are regulated on their respective cell types, and so it is difficult to speculate on how or whether these may be upregulated by the latent HIV-1 reservoir, involved in control of the latent reservoir, or may have another relationship to the reservoir. Future mechanistic studies in models of HIV-1 latency may help reveal what role NK cell interaction with these receptors may have in relation to HIV-1 persistence.

Several Killer-cell Immunoglobulin-like Receptors (KIRs) were also found by the LASSO to be predictive of HIV-1 persistence: KIR2DL1, KIR2DL3, and KIR2DS4. Within our cohort, we sought to provide some further support for these findings by performing genotyping on these KIR loci. We found that every one of our participants possessed at least one copy of KIR2DL1, and 47/50 of our participants possessed at least one copy of KIR2DL3. This suggests that the gene expression differences detected by the LASSO were not driven by the presence or absence of these genes but instead either by heterozygosity, allelic differences, or other effects that could change the frequency or level of expression of these proteins. For KIR2DS4, we were able to find a genetic counterpart to the protein level finding, confirming that the association found in our cohort between expression of KIR2DS4 and CA-DNA were being driven by genetic factors.

Clustering analysis followed by LASSO revealed that an NK cell phenotype that is CD16^+^ CD56^dim^ CD57^-^ LILRB1^-^ NKG2C^-^ has a negative correlation to CA-DNA. This finding was surprising because these markers denote an immature NK cell phenotype, while typically markers of NK maturity have been associated with better control of viral infections. CD57 has been associated with NK maturity (Lopez-Vergès et al., 2010) and increases in expression following several types of viral infections (Wilk and Blish, 2018). LILRB1 is similarly increased in viral infections, and has been associated with improved viral suppression *in vitro* (Scott-Algara et al., 2008). NKG2C expression marks the population of memory-like NK cells induced by CMV infection (Gumá et al., 2006; Lopez-Verges et al., 2011; Foley et al., 2012a; Foley et al., 2012b). CD57 and NKG2C expression have both been associated with specialization of function for ADCC, and reduced ability to perform other effector functions (Lopez-Vergès et al., 2010; Schlums et al., 2015). Interestingly, we found that although this population of cells expressed slightly lower levels of KIR, consistent with an immature NK cell population, it also expressed slightly lower levels of NKG2A, consistent with a mature NK cell population. It is therefore not as straightforward to classify these cells as into prior definitions of mature or immature NK cells. In one prior paper, CD57 expression on NK cells was found to correlate with increased NK cell diversity, which was in turn associated with increased rates of HIV-1 acquisition (Strauss-Albee et al., 2015). However, we found that there was no association between NK cell diversity and measurements of HIV persistence. The mechanism connecting this population to lower HIV persistence is therefore still unclear.

We also found an association between high surface expression of HLA-Bw6 and lower markers of HIV-1 persistence. Our genetic analysis revealed that this phenotype was driven by Bw4/Bw6 heterozygotes having the lowest measurements of HIV-1 persistence. This finding is important to consider in the context of the phenomenon of the post-treatment controller [reviewed in (Cockerham et al., 2016)]. These are rare individuals who have interrupted their ART and have subsequently been able to maintain viral control. These individuals are more likely to have started ART during the acute stage of HIV infection (Namazi et al., 2018), and are more likely to possess so-called “risk” alleles of HLA-B, which have been associated with less effective HIV control in other cohorts (Sáez-Cirión et al., 2013). These risk alleles are also classified as HLA-Bw6. However, it is not known whether heterozygosity with regards to HLA-Bw4/6 is associated with post-treatment control.

The amino acids that determine the Bw4 and Bw6 epitopes (Wan et al., 1986) form a part of the peptide binding groove (Garrett et al., 1989), and may be important in determining the T-cell-mediated responses to HIV-1. While peripheral blood T-cell ELISPOT reactivity to HIV-1 peptides showed no association to HLA-Bw4/6 genotype or to markers of HIV-1 persistence, we hypothesize that the reactivities important to mediating this heterozygote advantage may reside in secondary lymphoid organs, and not comprise a measurable component of the peripheral blood T-cell compartment. Prior work investigating the association of the Bw4/6 epitope with viral responses and T-cell responses has mostly focused on Bw4 or Bw6 homozygotes. Therefore, the association between Bw4/Bw6 heterozygosity and reduced HIV-1 persistence must be corroborated by study in a larger cohort.

Due to the n<<p regime of our regression studies, we were limited to the LASSO and statistical analyses without significance tests for most of our questions. Future studies following up on these findings should use lower parameter approaches focused on features selected by our approach in an independent cohort to validate these results. Furthermore, the most robust measurement for the HIV-1 reservoir that has been developed in recent years is the intact proviral DNA assay (IPDA) (Bruner et al., 2019), which is thought to best reflect the number of viruses in infected cells that may be able to reactivate upon interruption of ART. However, we were unable to obtain intact proviral DNA (IPDA) measurements on the majority of our participants, and so our analyses were limited to CA-DNA, CA-RNA, and SCA, which are more properly considered markers of HIV-1 persistence rather than direct measurements of the replication competent latent reservoir. Future work analyzing the relationship between the NK cell receptor-ligand repertoire should include IPDA as a primary assay to measure the latent HIV-1 reservoir. Other factors such as CMV coinfection can alter immune cell compartments in people living with HIV (Wijer Van de et al., 2021), and subclinical CMV replication is associated with higher HIV reservoirs (Christensen-Quick et al., 2019). CMV serostatus was not available on many of the participants in our cohort, but a previously published study from another subset of ACTG5321 participants with similar demographics to our cohort found that 94% of participants were CMV seropositive (Hodowanec et al., 2019). It would be interesting in future studies to examine the impact of CMV serostatus on the relationship between NK cell receptors and ligands and HIV persistence. Finally, events early in infection and at ART initiation may play a crucial role in the establishment and frequency of the latent HIV-1 reservoir (Abrahams et al., 2019). While we were able to corroborate some of our protein level findings with genetic data, suggesting that these expression differences were likely present both at the study timepoints and at the time of ART initiation, it is uncertain whether the associations we observed between protein expression on long-term ART and HIV-1 persistence reflect protein expression differences that would have been present at ART-initiation. Future work should include longitudinal studies that evaluate the immune phenotype of participants at timepoints prior to and shortly after ART initiation in relation to the later levels of latent HIV-1 reservoir or HIV-1 persistence.

In this work, we were able to uncover novel associations between markers of HIV-1 persistence and the NK cell receptor and ligand repertoire, which suggest a possible role for NK cells in regulating the latent reservoir. We found that an HLA-Bw4/6 heterozygous genotype is associated with lower markers of HIV-1 persistence, which we hypothesize to be due to a T-cell mediated effect in secondary lymphoid organs. These results provide a promising direction for future research into host factors driving the control of the latent HIV-1 reservoir.

## Data Availability Statement

The datasets presented in this article are not readily available due to ethical restrictions, however, they are available with the written agreement of the AIDS Clinical Trials Group (ACTG). Requests to access the datasets should be directed to Harvard Statistical and Data Analysis Center (SDAC), sdac.data@sdac.harvard.edu.

## Ethics Statement

The studies involving human participants were reviewed and approved by Stanford University Institutional Review Board. The patients/participants provided their written informed consent to participate in this study.

## Author Contributions

EV and CB conceptualized the initial project. EV, GI, TR, RV, GM-C, NZ, and MM contributed to experimental execution. GI and EV performed statistical analyses. GI, EV, SB, RB, SH, and CB contributed to the design of statistical analyses. GI wrote the first draft of the manuscript. GI, EV, GM-C, RG, RB, JE, MM, MC, and CB contributed substantially to editing and revision of the manuscript. RG, DM, JE, and JM are chairs of the ACTG 5321 trial and contributed to enrollment of the cohort. SB, MC, JM, RB, RG, SH, CB, JC, and RJ contributed to the supervision and project administration. All authors read and approved the manuscript for submission.

## Funding

Research reported in this publication was supported by the National Institute of Allergy and Infectious Diseases of the National Institutes of Health under Award Number UM1 AI068634, UM1 AI068636 and UM1 AI106701 for ACTG trial 5321. This project has been funded in part with federal funds from the Frederick National Laboratory for Cancer Research, under Contract No. HHSN261200800001E. This Research was supported in part by the Intramural Research Program of the NIH, Frederick National Lab, Center for Cancer Research. This work was supported by NIH/NIDA DP1DA04608903 to CB, NIH Ruth L. Kirschstein Institutional National Research Service Award 5T32AI007290 and the Stanford Graduate Fellowship to GI, ﻿NIH Ruth L. Kirschstein Institutional National Research Service Award T32AI007502 and TL1TR001084, and NIH/NIAID K08AI138640 to EV. CB is a BWF Investigator in the Pathogenesis of Infectious Diseases and an Investigator of the Chan Zuckerberg Biohub.

## Author Disclaimer

The content is solely the responsibility of the authors and does not necessarily represent the official views of the National Institutes of Health. The content of this publication does not necessarily reflect the views or policies of the Department of Health and Human Services, nor does mention of trade names, commercial products, or organizations imply endorsement by the U.S. Government.

## Conflict of Interest

The authors declare that the research was conducted in the absence of any commercial or financial relationships that could be construed as a potential conflict of interest.

## Publisher’s Note

All claims expressed in this article are solely those of the authors and do not necessarily represent those of their affiliated organizations, or those of the publisher, the editors and the reviewers. Any product that may be evaluated in this article, or claim that may be made by its manufacturer, is not guaranteed or endorsed by the publisher.
