# Supplementary material for: Natural Killer Cell Receptors and Ligands Are Associated With Markers of HIV-1 Persistence in Chronically Infected ART Suppressed Patients
Source: Front Cell Infect Microbiol. 2022 Feb 10;12:757846. doi: 10.3389/fcimb.2022.757846 (PMC8866573; doi:10.3389/fcimb.2022.757846)
Supplement: Supplementary file 1 [file DataSheet_1.pdf]

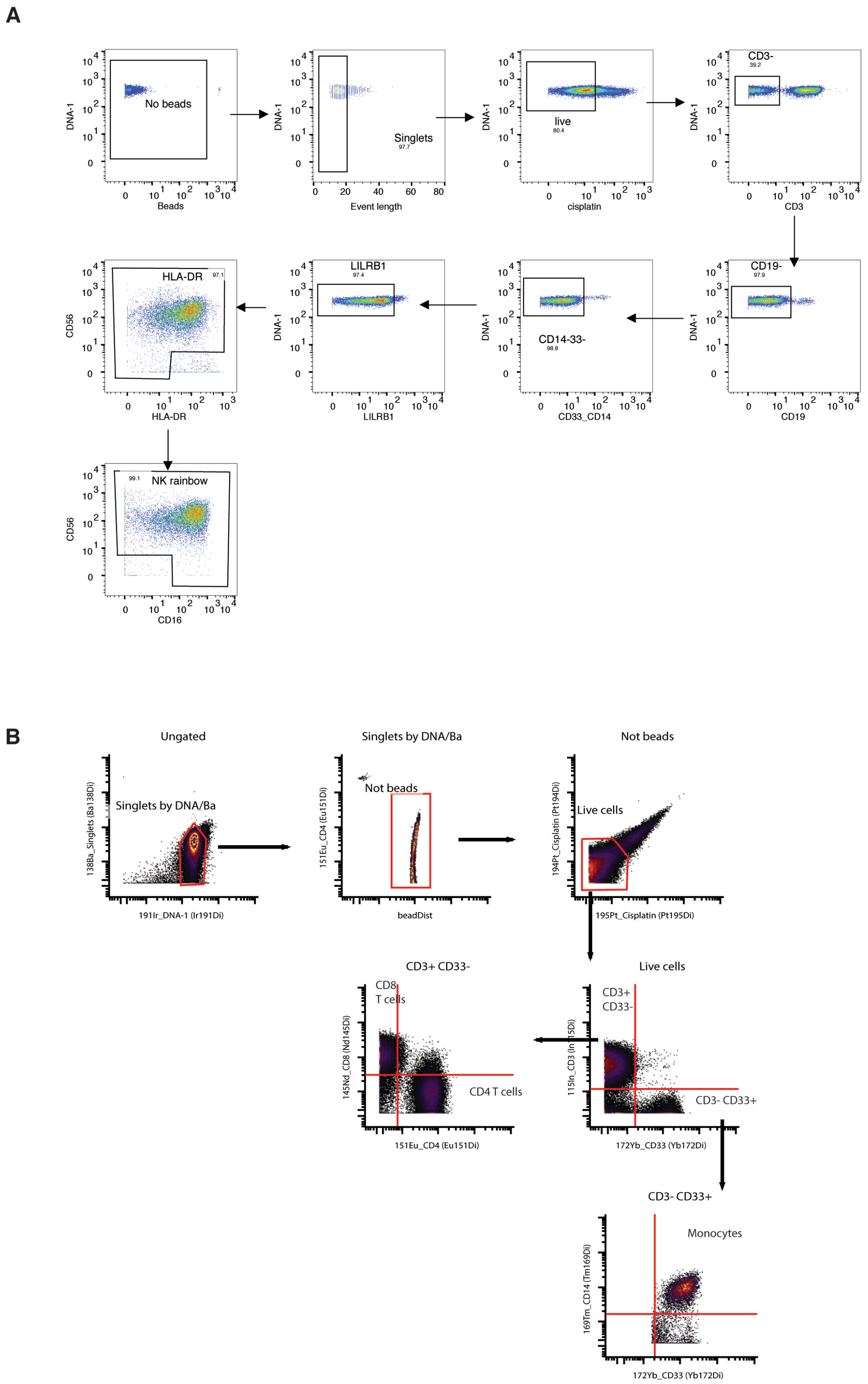

Supplemental figure 1. **Gating schemes.** (A) Gating scheme for NK cells (B) Gating scheme for CD4 T-cells, CD8 T-cells, and Monocytes
