# Supplementary material for: Natural Killer Cell Receptors and Ligands Are Associated With Markers of HIV-1 Persistence in Chronically Infected ART Suppressed Patients
Source: Front Cell Infect Microbiol. 2022 Feb 10;12:757846. doi: 10.3389/fcimb.2022.757846 (PMC8866573; doi:10.3389/fcimb.2022.757846)
Supplement: Supplementary file 2 [file DataSheet_2.pdf]

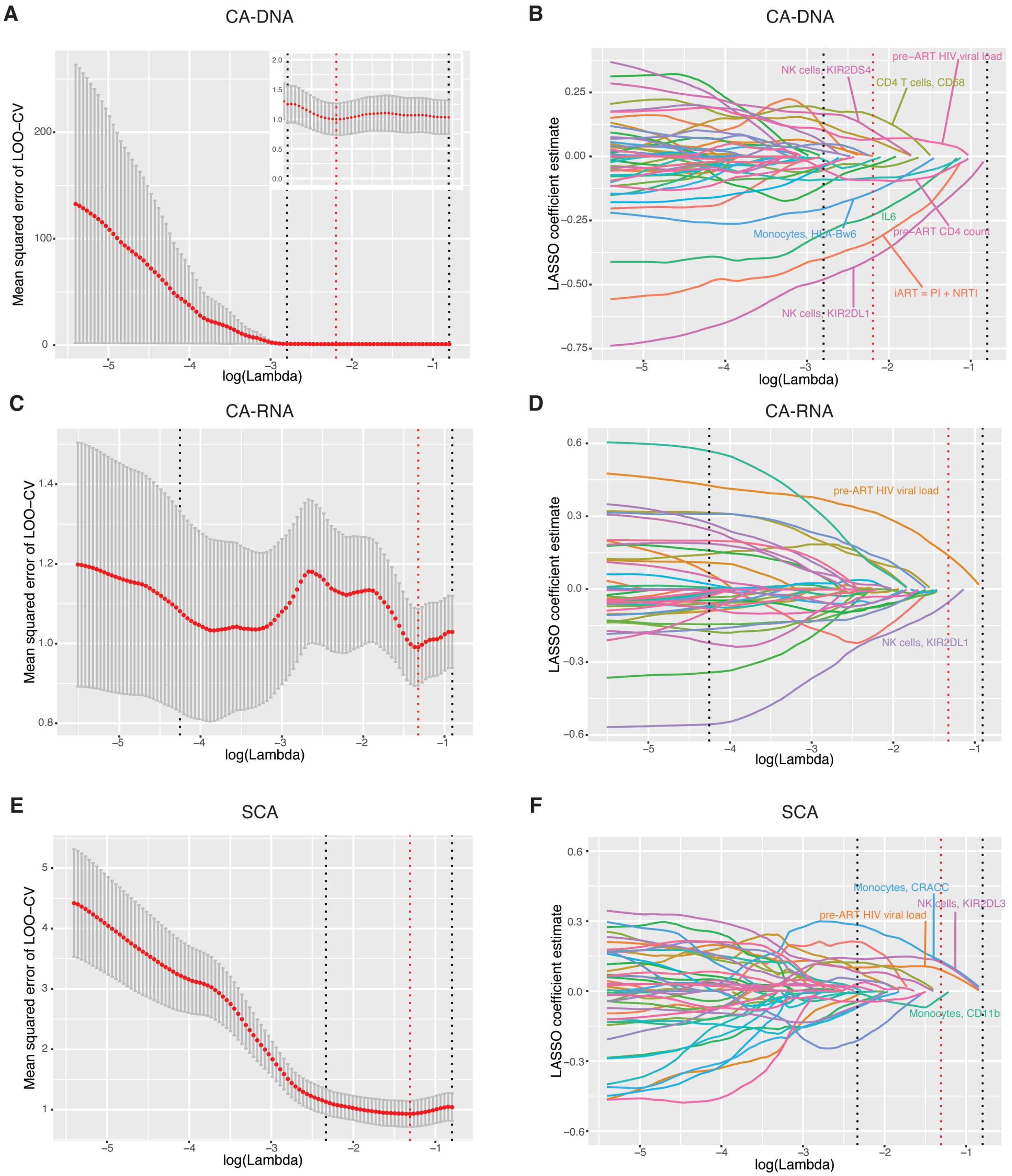

Supplemental Figure 2. **LASSO optimization** (A, C, E) Plots showing the cross-validated overfitting error (calculated by leave-one-out cross validation) at a range of LASSO stringencies, predicting (A) CA-DNA, (C) CA-RNA, or (E) SCA. The red dashed line indicates the stringency with the minimum overfitting error, while the black dashed lines indicate the range of stringencies within a standard error of the minimum. (B, D, F) Plots showing the estimated coefficients for all the variables at the full range of stringencies, with labels for variables with large coefficients at the optimal stringency, for the model predicting (B) CA-DNA, (D) CA-RNA, or (F) SCA.
