# Supplementary material for: Natural Killer Cell Receptors and Ligands Are Associated With Markers of HIV-1 Persistence in Chronically Infected ART Suppressed Patients
Source: Front Cell Infect Microbiol. 2022 Feb 10;12:757846. doi: 10.3389/fcimb.2022.757846 (PMC8866573; doi:10.3389/fcimb.2022.757846)
Supplement: Supplementary file 3 [file DataSheet_3.pdf]

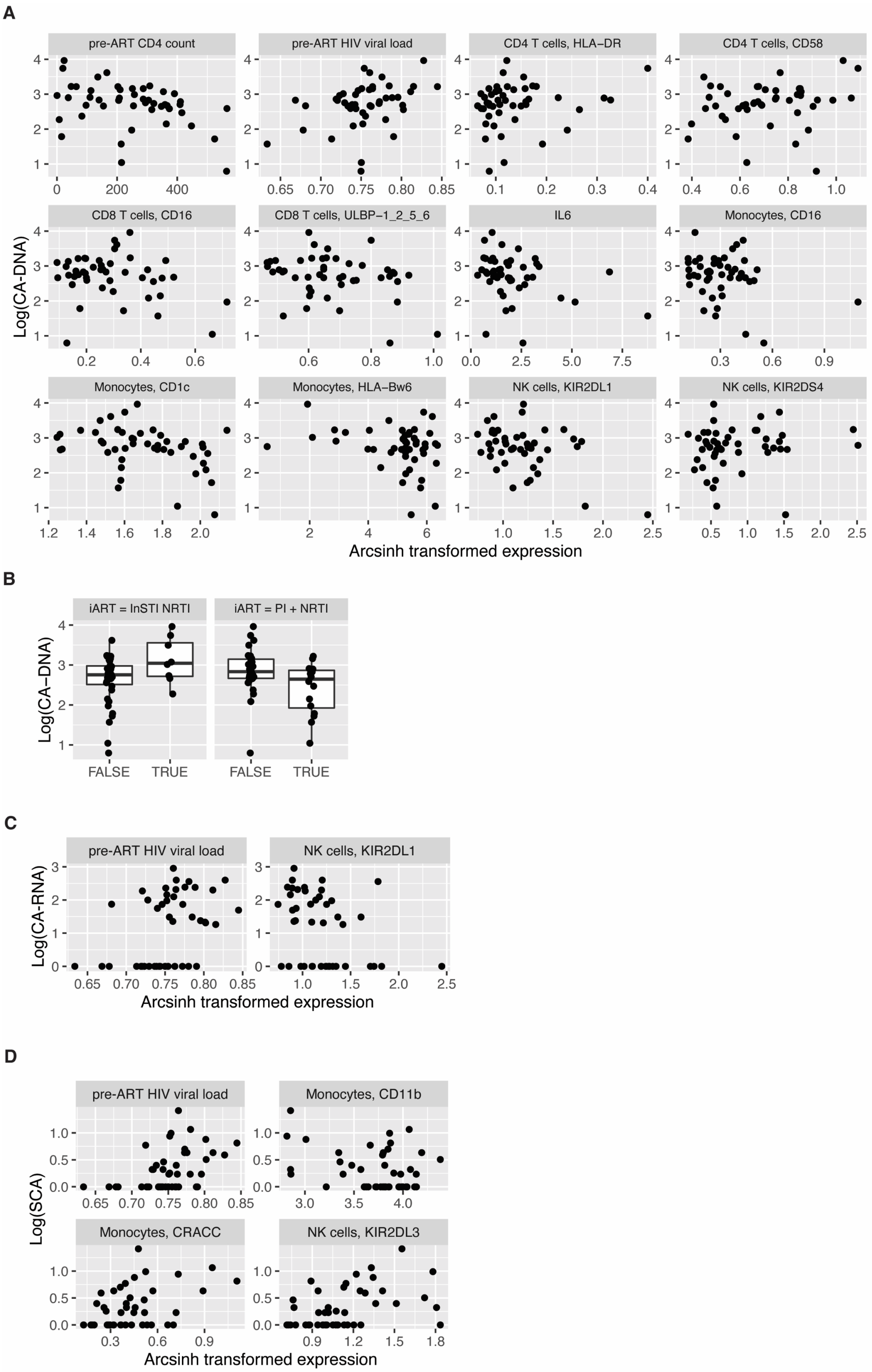

Supplemental figure 3. **Associations between variables selected by the LASSO and CA-DNA, CA-RNA, or SCA.** (A) Scatterplots showing variables selected by LASSO for CA-DNA. (B) Boxplots showing variables selected by LASSO for CA-DNA. (C) Scatterplots showing variables selected by LASSO for CA-RNA. (D) Scatterplots showing variables selected by LASSO for SCA.
