# Supplementary material for: Natural Killer Cell Receptors and Ligands Are Associated With Markers of HIV-1 Persistence in Chronically Infected ART Suppressed Patients
Source: Front Cell Infect Microbiol. 2022 Feb 10;12:757846. doi: 10.3389/fcimb.2022.757846 (PMC8866573; doi:10.3389/fcimb.2022.757846)
Supplement: Supplementary file 4 [file DataSheet_4.pdf]

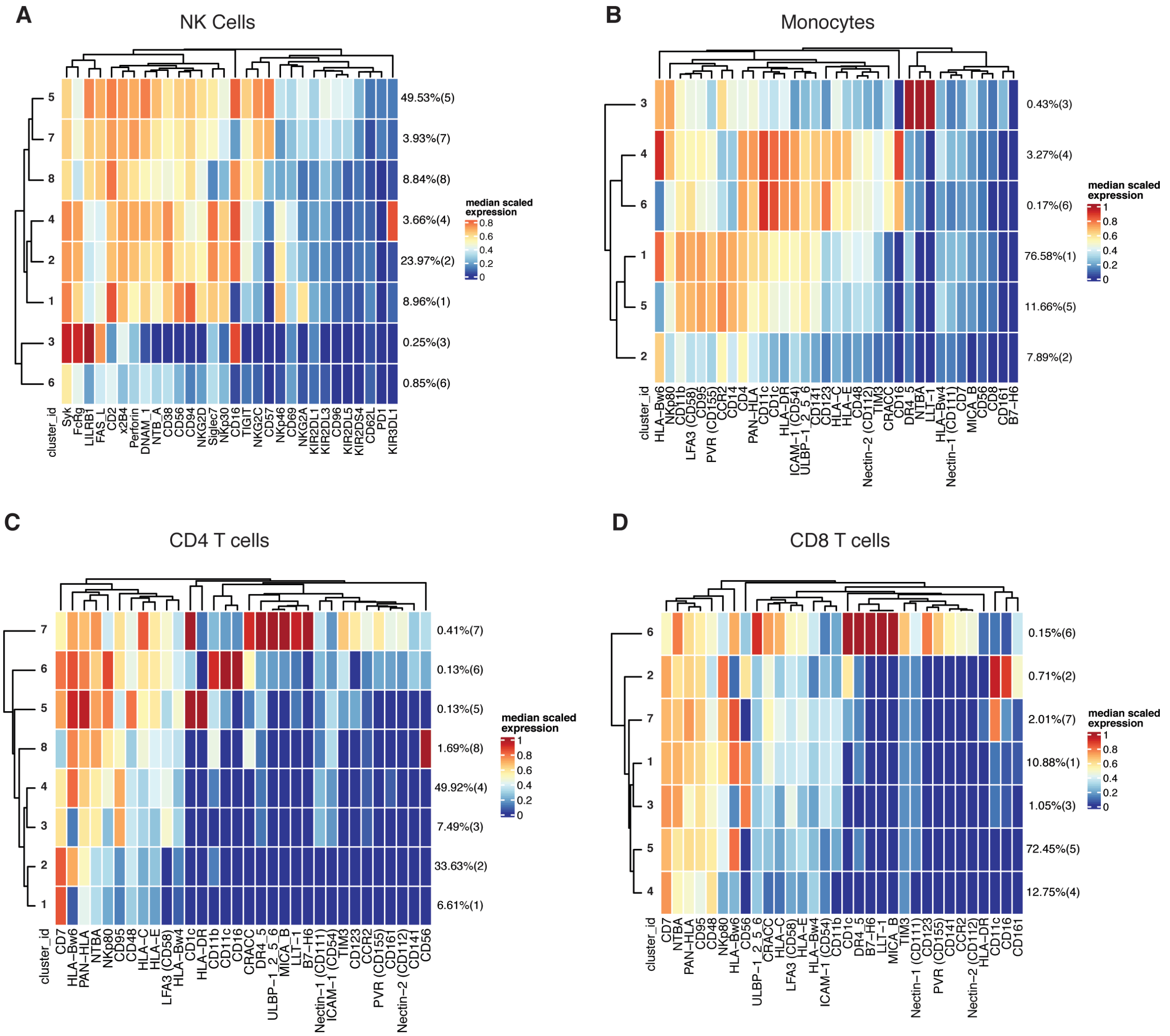

Supplemental figure 4. **Results of FlowSOM clustering with ConsensusClusterPlus metaclustering of NK cells, CD4 T-cells, CD8 T-cells, and Monocytes.** (A-D) Heatmaps showing clusters as rows and scaled median expression of each marker as columns. Marker expression is shown as the median expression of cells in each cluster, relative to the total range of expression values of that marker within all cells. To the right of the heatmap, the percentage of total cells belonging to each cluster is displayed. Heatmaps are shown for (A) NK cells, (B) Monocytes, (C) CD4 T-cells, and (D) CD8 T-cells.
