# Supplementary figures and images for: Natural Killer Cell Receptors and Ligands Are Associated With Markers of HIV-1 Persistence in Chronically Infected ART Suppressed Patients
Source: Front Cell Infect Microbiol. 2022 Feb 10;12:757846. doi: 10.3389/fcimb.2022.757846 (PMC8866573; doi:10.3389/fcimb.2022.757846)

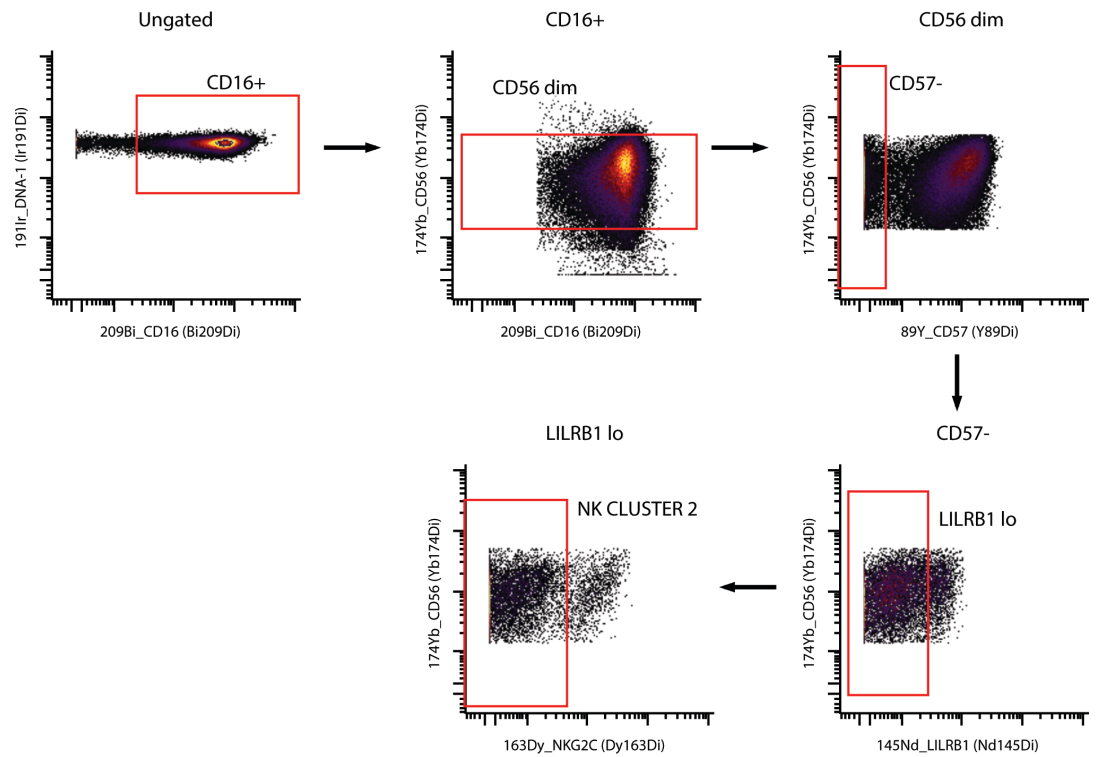

Supplemental figure 5. **Gating scheme for low dimensional gating approximation of NK cluster 2.**

Supplement: Supplementary file 5 [file DataSheet_5.pdf]
