# Supplementary material for: Natural Killer Cell Receptors and Ligands Are Associated With Markers of HIV-1 Persistence in Chronically Infected ART Suppressed Patients
Source: Front Cell Infect Microbiol. 2022 Feb 10;12:757846. doi: 10.3389/fcimb.2022.757846 (PMC8866573; doi:10.3389/fcimb.2022.757846)
Supplement: Supplementary file 6 [file DataSheet_6.pdf]

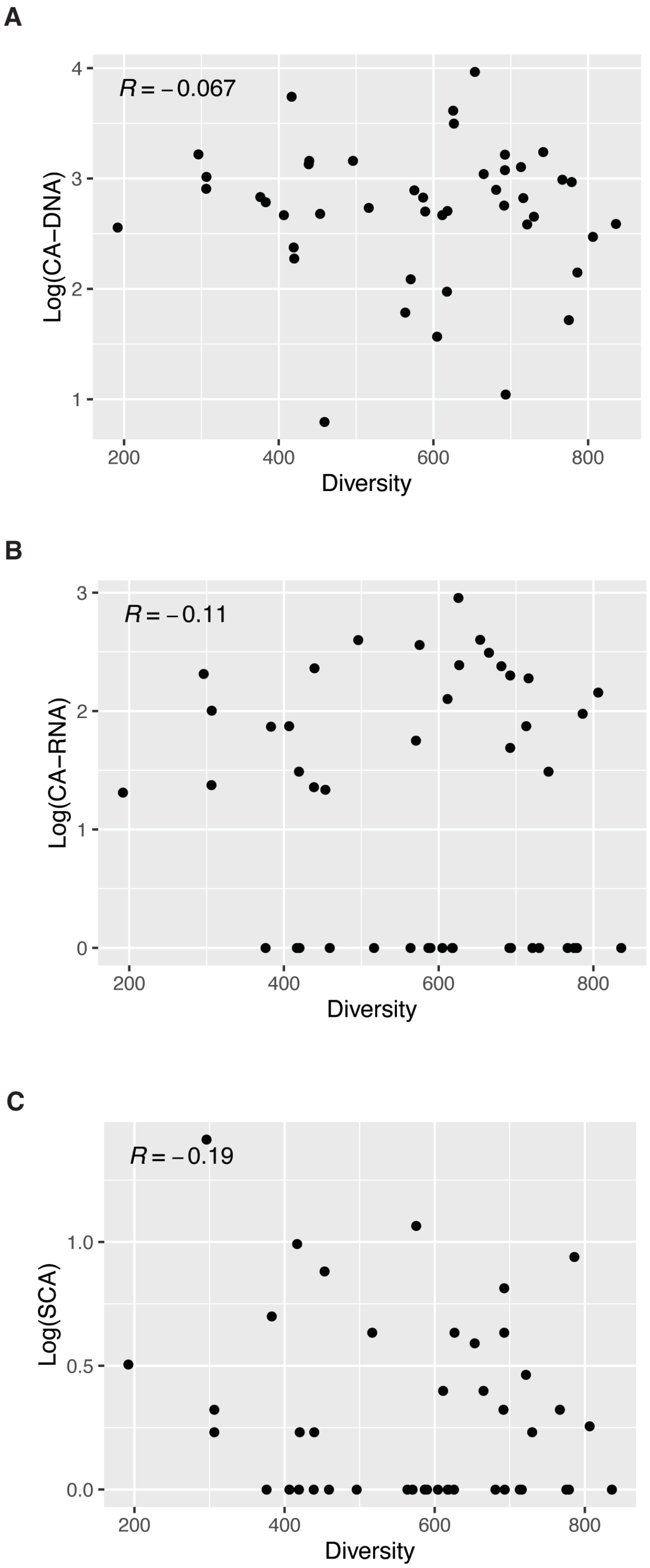

Supplemental figure 6. **No association found between measurements of HIV persistence and NK cell diversity.** Scatterplots showing the relationship between NK cell diversity and (A) CA-DNA, (B) CA-RNA, and (C) SCA.
