# Supplementary material for: Natural Killer Cell Receptors and Ligands Are Associated With Markers of HIV-1 Persistence in Chronically Infected ART Suppressed Patients
Source: Front Cell Infect Microbiol. 2022 Feb 10;12:757846. doi: 10.3389/fcimb.2022.757846 (PMC8866573; doi:10.3389/fcimb.2022.757846)
Supplement: Supplementary file 7 [file DataSheet_7.pdf]

A

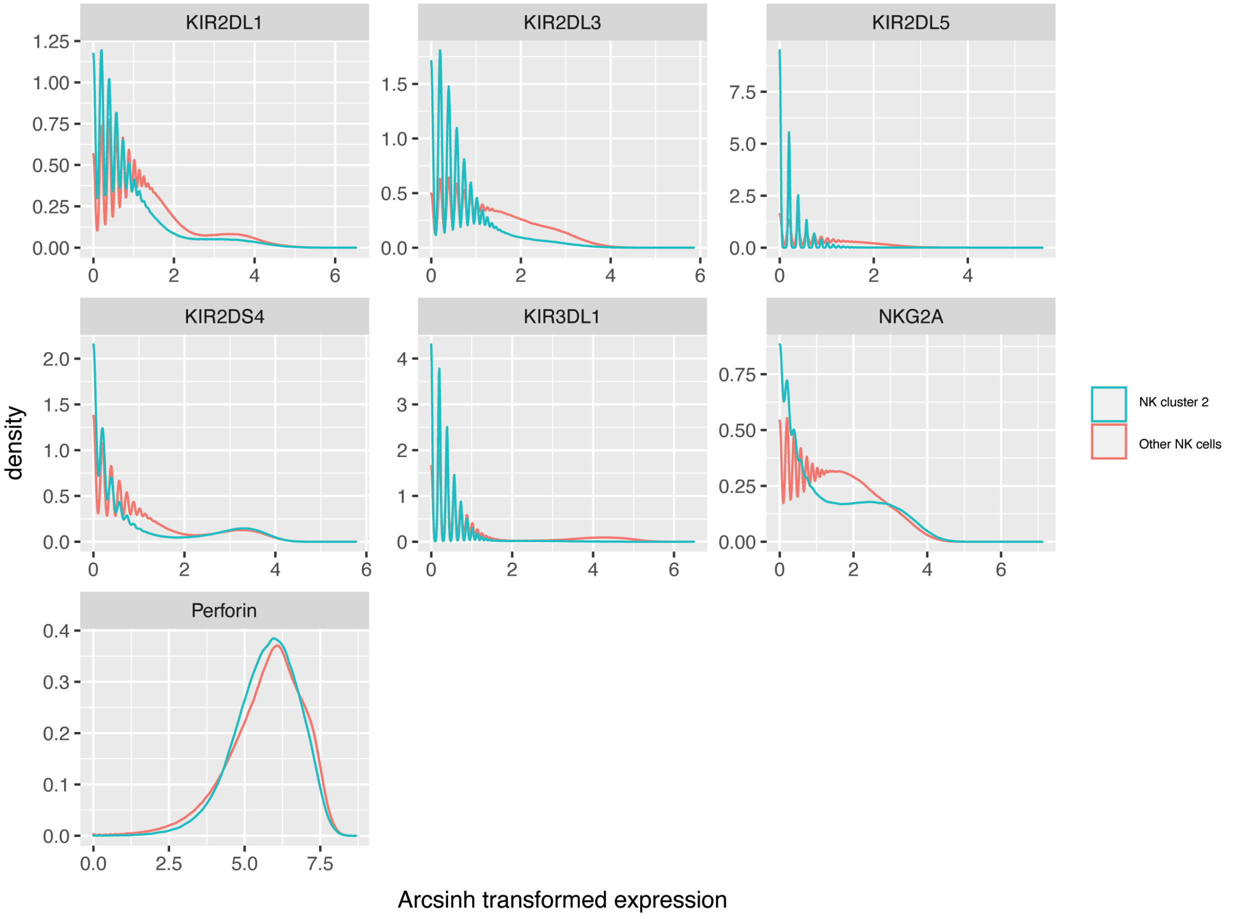

B

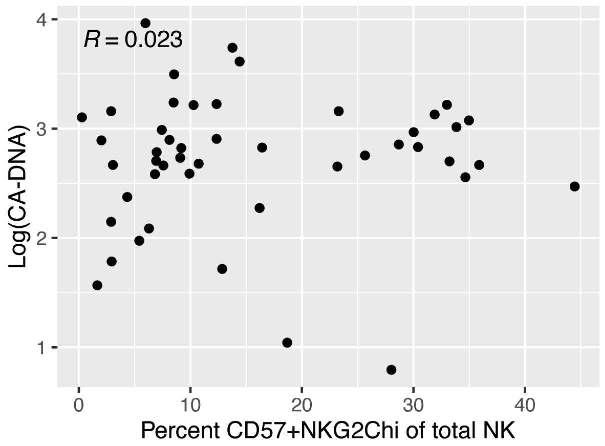

Supplemental figure 7. **Investigation of NK cell maturity.** (A) Histograms showing the phenotype of NK cluster 2 compared to all other NK cells. (B) Scatterplot showing the relationship between gated frequency of $CD57^{+}NKG2C^{hi}$ NK cells and CA-DNA.
