# Supplementary material for: Natural Killer Cell Receptors and Ligands Are Associated With Markers of HIV-1 Persistence in Chronically Infected ART Suppressed Patients
Source: Front Cell Infect Microbiol. 2022 Feb 10;12:757846. doi: 10.3389/fcimb.2022.757846 (PMC8866573; doi:10.3389/fcimb.2022.757846)
Supplement: Supplementary file 8 [file DataSheet_8.pdf]

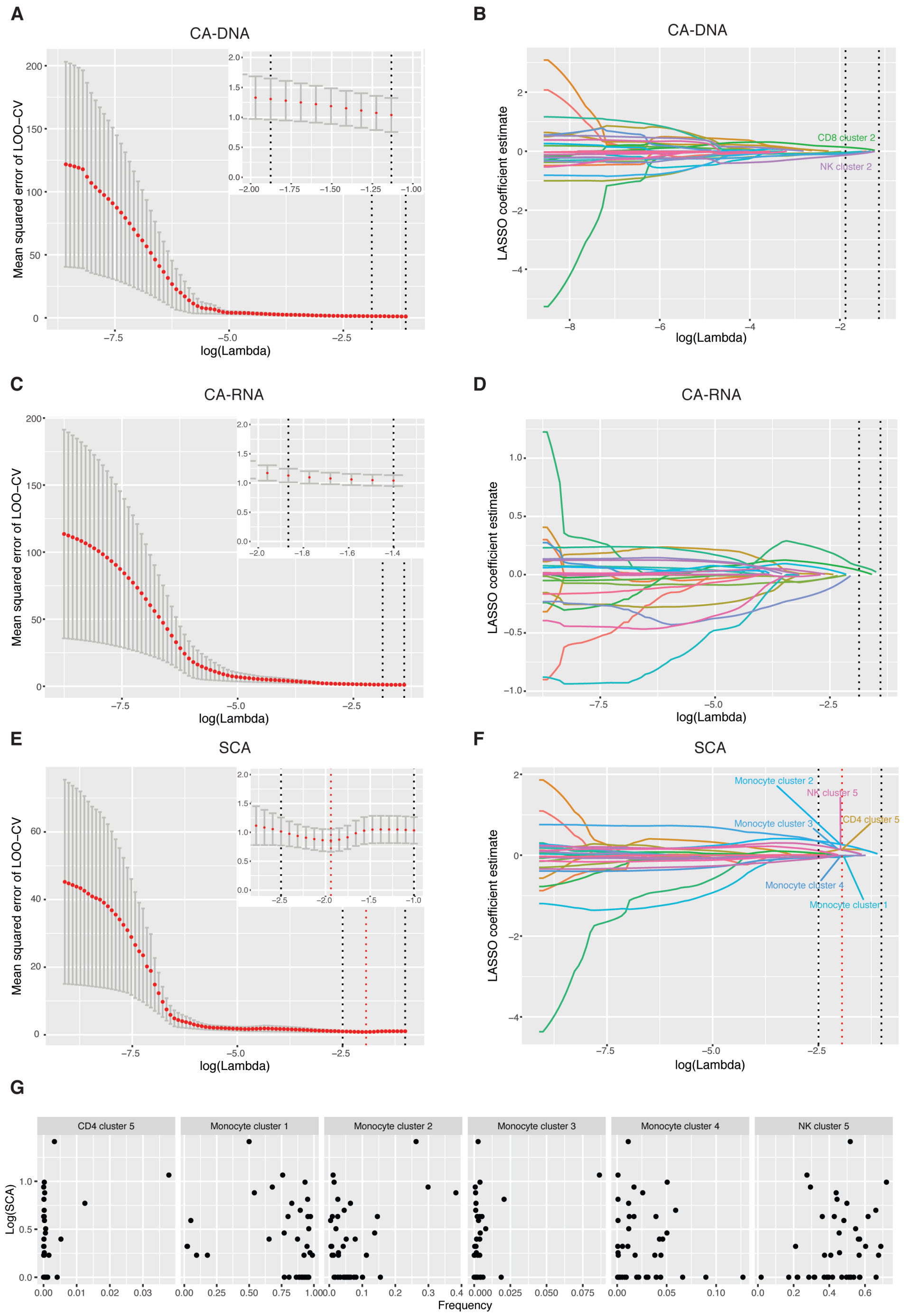

Supplemental figure 8. **FlowSOM and LASSO analyses show weak association between flowSOM clusters and CA-RNA or SCA.** (A, C, E) Plots showing the cross validated overfitting error at a range of LASSO stringencies, predicting (A) CA-DNA (n = 47), (C) CA-RNA (n = 46), or (E) SCA (n = 45) with cluster frequency. The black dashed lines indicate the range of stringencies within a standard error of the minimum, and the red dashed line indicates the minimum overfitting error in (E). (B, D, F) Plots showing the estimated coefficients for each cluster at the full range of stringencies for (B) CA-DNA, (D) CA-RNA, or (F) SCA. No coefficients were selected in (D), while labels are shown for all clusters selected in (F). (G) Scatterplots showing the associations between SCA and the variables selected by the LASSO.
