# Supplementary material for: Natural Killer Cell Receptors and Ligands Are Associated With Markers of HIV-1 Persistence in Chronically Infected ART Suppressed Patients
Source: Front Cell Infect Microbiol. 2022 Feb 10;12:757846. doi: 10.3389/fcimb.2022.757846 (PMC8866573; doi:10.3389/fcimb.2022.757846)
Supplement: Supplementary file 9 [file DataSheet_9.pdf]

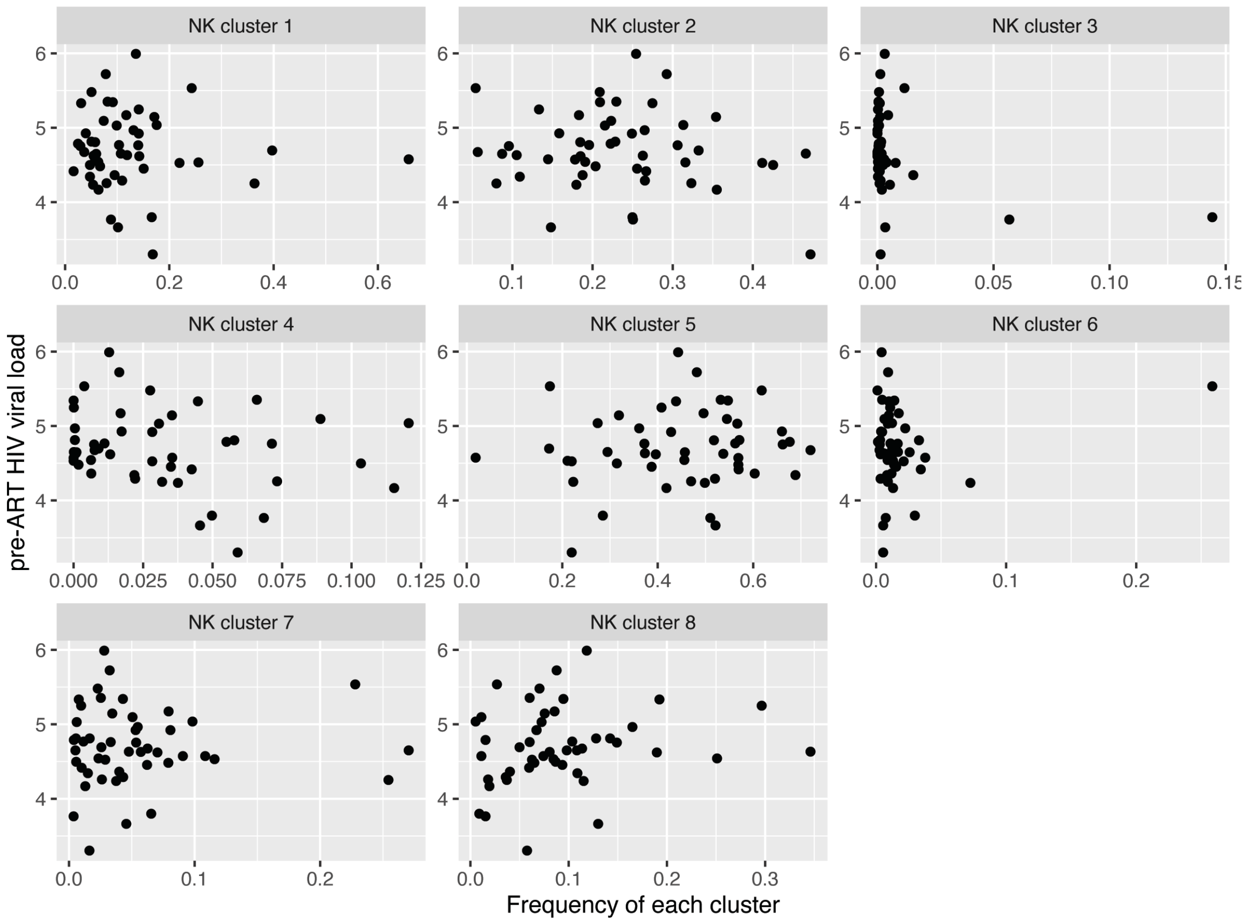

Supplemental figure 9. **Relationship between NK cell clusters and pre-ART HIV viral load.** Scatterplots showing the relationship between the frequency of each of the NK cell metaclusters and pre-ART HIV viral load.
