# Supplementary material for: Natural Killer Cell Receptors and Ligands Are Associated With Markers of HIV-1 Persistence in Chronically Infected ART Suppressed Patients
Source: Front Cell Infect Microbiol. 2022 Feb 10;12:757846. doi: 10.3389/fcimb.2022.757846 (PMC8866573; doi:10.3389/fcimb.2022.757846)
Supplement: Supplementary file 10 [file DataSheet_10.pdf]

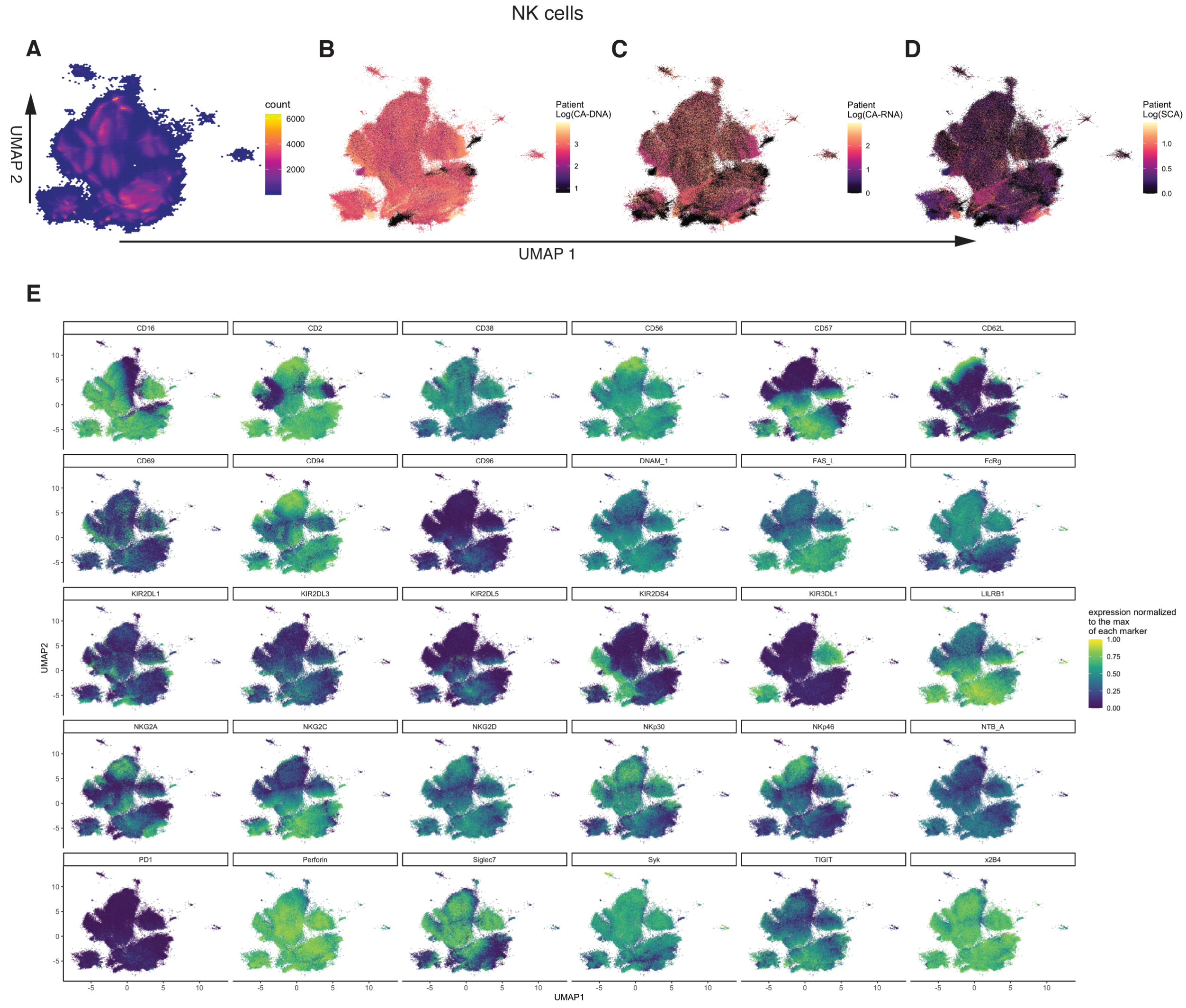

Supplemental figure 10. **UMAP of NK cells.** (A) Colored by density (B-D) Colored by reservoir measurements at first timepoint (E) Colored by NK receptors and phenotyping markers
