# Supplementary material for: Natural Killer Cell Receptors and Ligands Are Associated With Markers of HIV-1 Persistence in Chronically Infected ART Suppressed Patients
Source: Front Cell Infect Microbiol. 2022 Feb 10;12:757846. doi: 10.3389/fcimb.2022.757846 (PMC8866573; doi:10.3389/fcimb.2022.757846)
Supplement: Supplementary file 13 [file DataSheet_13.pdf]

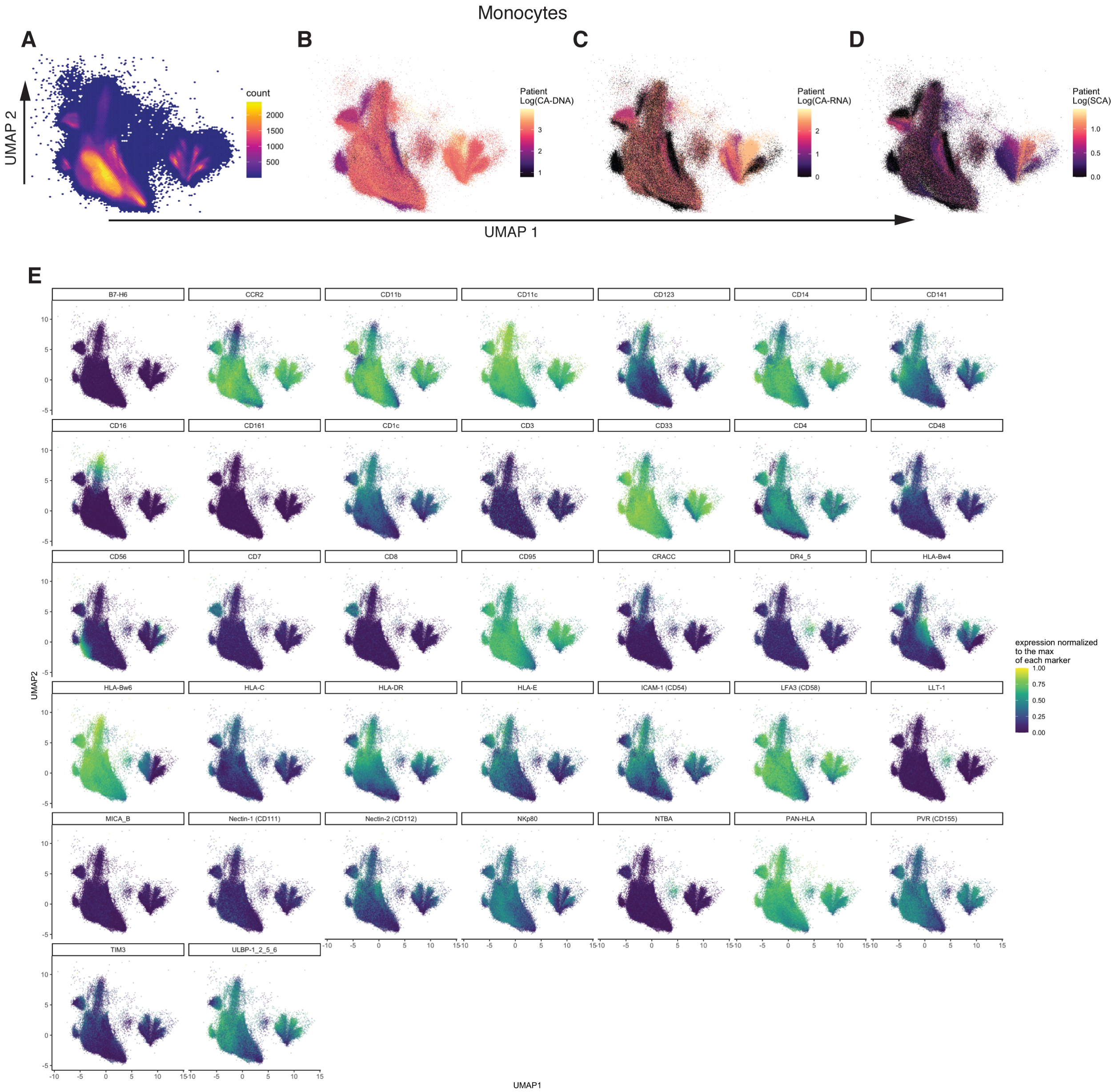

Supplemental figure 13. **UMAP of Monocytes.** (A) Colored by density (B-D) Colored by reservoir measurements at first timepoint (E) Colored by NK ligands and phenotyping markers
