# Supplementary material for: Natural Killer Cell Receptors and Ligands Are Associated With Markers of HIV-1 Persistence in Chronically Infected ART Suppressed Patients
Source: Front Cell Infect Microbiol. 2022 Feb 10;12:757846. doi: 10.3389/fcimb.2022.757846 (PMC8866573; doi:10.3389/fcimb.2022.757846)
Supplement: Supplementary file 14 [file DataSheet_14.pdf]

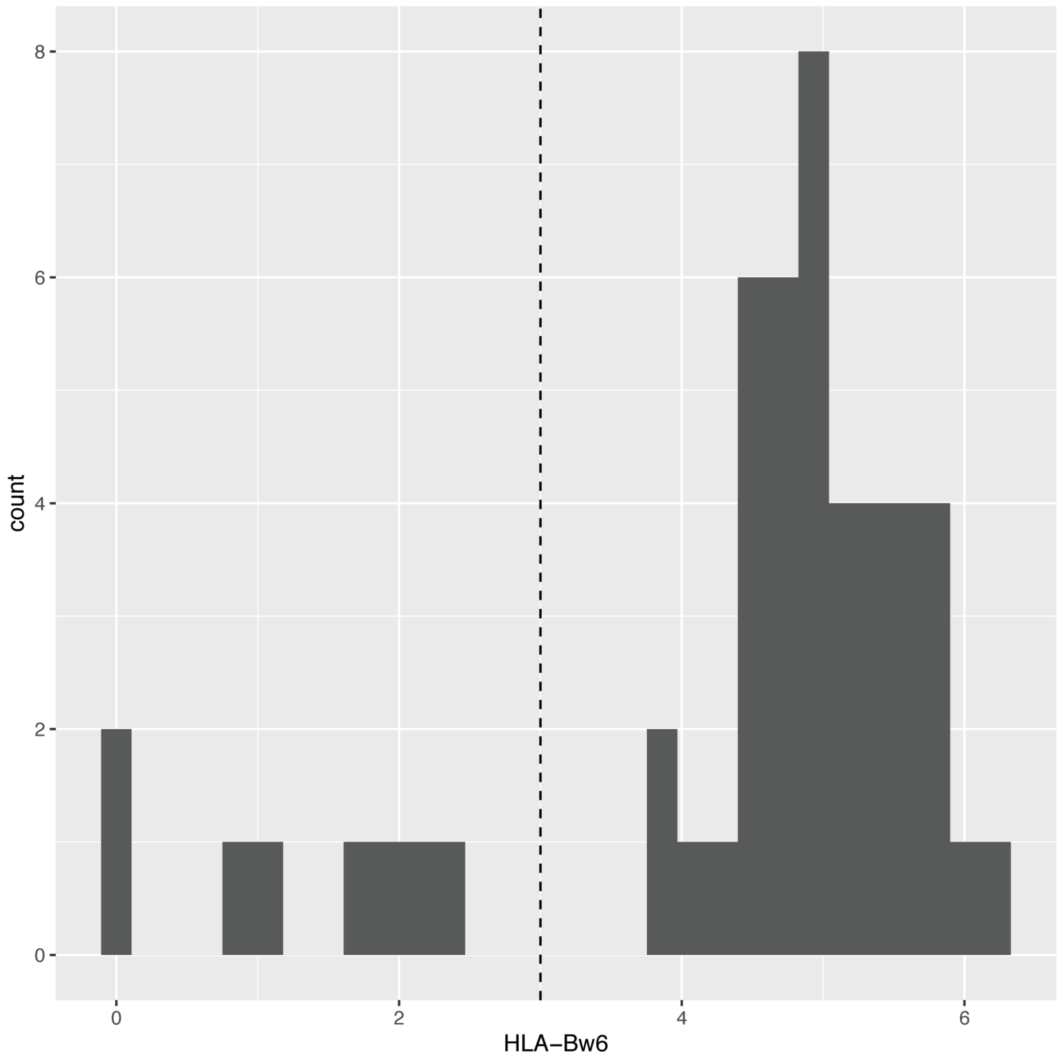

Supplemental figure 14. **Cutoff used to define HLA-Bw6 hi/low.** A histogram shows the distribution of mean Bw6 expression on CD4 T-cells at entry. The dashed line represents the cutoff used to define Bw6 hi vs. low.
