# Supplementary material for: Natural Killer Cell Receptors and Ligands Are Associated With Markers of HIV-1 Persistence in Chronically Infected ART Suppressed Patients
Source: Front Cell Infect Microbiol. 2022 Feb 10;12:757846. doi: 10.3389/fcimb.2022.757846 (PMC8866573; doi:10.3389/fcimb.2022.757846)
Supplement: Supplementary file 15 [file DataSheet_15.pdf]

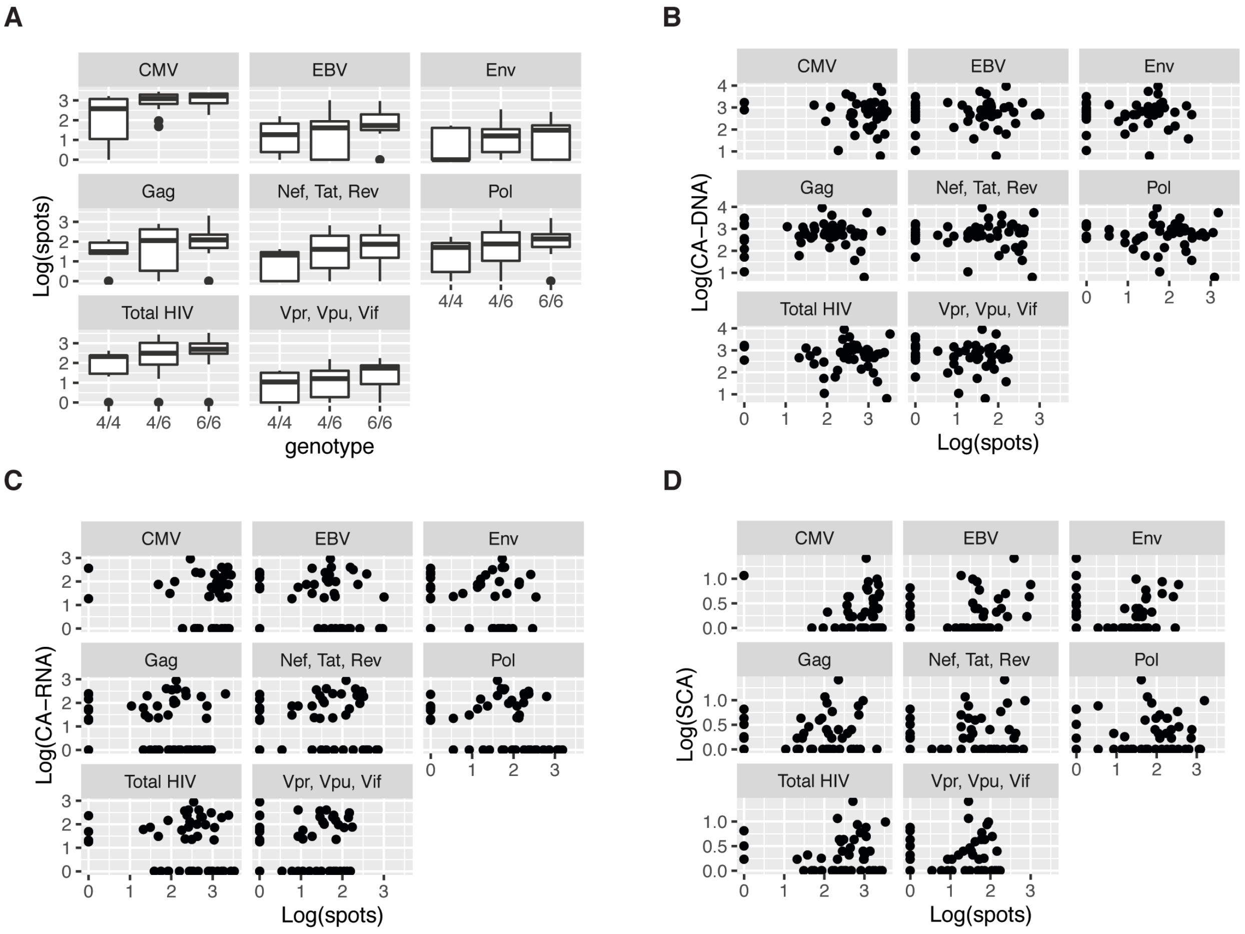

Supplemental figure 15. **Associations between ELISPOT reactivity and Bw4/6 genotype, the latent reservoir.** (A) Boxplots showing minimal association between Bw4/6 genotype and ELISPOT peptide reactivities. (B-D) Scatterplots showing minimal association between ELISPOT peptide reactivities and (B) CA-DNA, (C) CA-RNA, (D) SCA.
